# Supplementary material for: Tobacco control policies on cancer prevention in the Eastern Mediterranean Region, 2025–2050: A modeling study
Source: PLoS Med. 2026 Apr 24;23(4):e1005032. doi: 10.1371/journal.pmed.1005032 (PMC13108767; doi:10.1371/journal.pmed.1005032)
Supplement: S1 Table — (DOCX) [file pmed.1005032.s001.docx]

**S1 Table:** Gender-specific Tobacco smoking prevalence, MPOWER index score, cigarette affordability index, and literacy rate for each EMR country

| Country | Tobacco prevalence in 2025 (%) | | MPOWER implementation score (range 7 - 34) | Tobacco Affordability Index | Literacy rate 2022 (%) | |
| --- | --- | --- | --- | --- | --- | --- |
|  | **Men (%)** | **Women (%)** | **2022** | **2020** | **Men** | **Women** |
| Afghanistan | 14.6 | 1.6 | 20 | 11.1 | 52.1 | 22.6 |
| Bahrain | 23.8 | 4.9 | 26 | 2.1 | 98.8 | 96.1 |
| Egypt | 51.2 | 0.3 | 27 | 3.0 | 80.0 | 68.9 |
| Iran | 15.7 | 0.9 | 31 | 9.5 | 93.0 | 84.8 |
| Iraq | 36.9 | 1.4 | 26 | 0.8 | 91.2 | 79.9 |
| Jordan | 58.4 | 14.1 | 30 | 7.1 | 98.7 | 98.1 |
| Kuwait | 33.0 | 2.0 | 22 | 0.7 | 97.1 | 95.3 |
| Lebanon | 43.3 | 25.7 | 25 | 5.0 | 91.8 | 82.8 |
| Morocco | 23.6 | 0.9 | 24 | 6.1 | 85.6 | 69.1 |
| Oman | 15.3 | 0.3 | 20 | 2.5 | 98.6 | 92.7 |
| Pakistan | 22.5 | 2.6 | 26 | 3.4 | 69.3 | 46.5 |
| Qatar | 19.9 | 1.9 | 27 | 0.7 | 93.1 | 94.7 |
| Saudi Arabia | 26.9 | 1.8 | 29 | 2.6 | 98.6 | 98.6 |
| Tunisia | 37.6 | 1.4 | 25 | 3.3 | 87.7 | 73.7 |
| United Arab Emirates | 13.9 | 2.5 | 25 | 1.1 | 98.8 | 97.6 |
| Yemen | 27.2 | 5.9 | 22 | 45.0 | 73.0 | 35.0 |

Smoking prevalence represents the percentage of current tobacco smokers among adults in 2025, stratified by sex. MPOWER implementation score (range 7–34) reflects the level of implementation of the six WHO MPOWER tobacco control measures in 2022, with higher scores indicating stronger policy implementation. The tobacco affordability index (2020) represents the relative affordability of cigarettes, calculated based on price relative to income; higher values indicate greater affordability. Literacy rate (2022) represents the percentage of literate adults aged ≥15 years, stratified by sex. EMR = Eastern Mediterranean Region.

Data sources included WHO Global Health Observatory, WHO MPOWER reports, and World Bank indicators (see Methods for detailed sources).
