# Supplementary material for: Tobacco control policies on cancer prevention in the Eastern Mediterranean Region, 2025–2050: A modeling study
Source: PLoS Med. 2026 Apr 24;23(4):e1005032. doi: 10.1371/journal.pmed.1005032 (PMC13108767; doi:10.1371/journal.pmed.1005032)
Supplement: S2 Table — (DOCX) [file pmed.1005032.s002.docx]

**S2 Table:** The association between change in MPOWER, affordability index, literacy rate, and prevalence of tobacco smoking in the EMR countries using historical data from 2010 to 2020

|  | Model A | | R^2^ | | |  | Model B | | R^2^ | | |
| --- | --- | --- | --- | --- | --- | --- | --- | --- | --- | --- | --- |
| **Men** | β coefficient (95% CI) | P-value | Within | Between | Overall |  | β coefficient (95% CI) | P-value | Within | Between | Overall |
| MPOWER | -0.26 (-0.42, -0.10) | 0.001 | 0.122 | 0.143 | 0.068 |  | -0.15 (-0.31, -0.001) | 0.049 | 0.275 | 0.0811 | 0.596 |
| Affordability | -0.12 (-0.22, -0.17) | 0.022 | 0.064 | 0.0004 | 0.0001 |  | -0.08 (-0.17, 0.01) | 0.100 | 0.275 | 0.0811 | 0.596 |
| Literacy rate | -0.27 (-0.38, -0.14) | 0.001 | 0.20 | 0.048 | 0.039 |  | -0.21 (-0.33, -0.09) | 0.001 | 0.275 | 0.0811 | 0.596 |
| **Women** |  |  |  |  |  |  |  |  |  |  |  |
| MPOWER | -0.12 (-0.17, -0.07) | 0.001 | 0.234 | 0.014 | 0.005 |  | -0.11 (-0.16, -0.06) | 0.001 | 0.311 | 0.027 | 0.008 |
| Affordability | -0.06 (-0.09, -0.02) | 0.001 | 0.132 | 0.012 | 0.004 |  | -0.04 (-0.07, -0.01) | 0.005 | 0.311 | 0.027 | 0.008 |
| Literacy rate | -0.007 (-0.02, 0.006) | 0.282 | 0.015 | 0.003 | 0.002 |  | -0.002 (-0.01, 0.01) | 0.707 | 0.311 | 0.027 | 0.008 |

*β coefficients were estimated using fixed-effects panel regression models including country (location) and year fixed effects, based on country-level data from 2010 to 2020. The dependent variable was the change in sex-specific smoking prevalence. Independent variables included changes in MPOWER implementation score, tobacco affordability index, and literacy rate.

*Model A presents unadjusted associations for each predictor separately. Model B represents the fully adjusted multiple model in which all predictors were entered simultaneously. Each coefficient represents the change in smoking prevalence (percentage points) associated with a one-unit increase in the corresponding policy indicator.

*R² statistics are reported as within-country, between-country, and overall model fit measures. P-values correspond to two-sided tests with statistical significance defined as P < 0.05. CI = 95% confidence interval. EMR = Eastern Mediterranean Region.
