# Supplementary material for: Tobacco control policies on cancer prevention in the Eastern Mediterranean Region, 2025–2050: A modeling study
Source: PLoS Med. 2026 Apr 24;23(4):e1005032. doi: 10.1371/journal.pmed.1005032 (PMC13108767; doi:10.1371/journal.pmed.1005032)
Supplement: S3 Table — (DOCX) [file pmed.1005032.s003.docx]

**S3 Table:** Gender -specific Current prevalence of tobacco smoking, prevalence of tobacco smoking with the highest MPOWER, prevalence with a-10 unit increase in affordability index, prevalence with maximizing literacy rate, and prevalence of tobacco smoking under all-policies combined scenario in each EMR country

| Country |  | **Men** | | | |  |  | **Women** | | | |
| --- | --- | --- | --- | --- | --- | --- | --- | --- | --- | --- | --- |
|  | **Current** | **MPOWER** | **Affordability** | **Literacy** | **All** |  | **Current** | **MPOWER** | **Affordability** | **Literacy** | **All** |
| Afghanistan | 14.6 | 11.0 | 13.4 | 1.7 | 1.2 |  | 1.6 | 0.0 | 1.0 | 1.6 | 0.0 |
| Bahrain | 23.8 | 21.7 | 22.6 | 23.5 | 21.5 |  | 4.9 | 3.9 | 4.3 | 4.9 | 3.6 |
| Egypt | 51.2 | 49.4 | 50.0 | 45.8 | 45.0 |  | 0.3 | 0.0 | 0.0 | 0.3 | 0.0 |
| Iran | 15.7 | 14.9 | 14.5 | 13.8 | 12.9 |  | 0.9 | 0.5 | 0.3 | 0.9 | 0.1 |
| Iraq | 36.9 | 34.8 | 35.7 | 34.5 | 32.9 |  | 1.4 | 0.4 | 0.8 | 1.4 | 0.1 |
| Jordan | 58.4 | 57.4 | 57.2 | 58.0 | 56.7 |  | 14.1 | 13.6 | 13.5 | 14.1 | 13.2 |
| Kuwait | 33.0 | 29.9 | 31.8 | 32.2 | 29.7 |  | 2.0 | 0.5 | 1.4 | 2.0 | 0.2 |
| Lebanon | 43.3 | 41.0 | 42.1 | 41.1 | 39.3 |  | 25.7 | 24.6 | 25.1 | 25.7 | 24.2 |
| Morocco | 23.6 | 21.0 | 22.4 | 19.7 | 18.1 |  | 0.9 | 0.0 | 0.3 | 0.9 | 0.0 |
| Oman | 15.3 | 11.7 | 14.1 | 14.9 | 12.0 |  | 0.3 | 0.0 | 0.0 | 0.3 | 0.0 |
| Pakistan | 22.5 | 20.4 | 21.3 | 14.2 | 13.8 |  | 2.6 | 1.6 | 2.0 | 2.6 | 1.3 |
| Qatar | 19.9 | 18.1 | 18.7 | 18.0 | 16.5 |  | 1.9 | 1.0 | 1.3 | 1.9 | 0.7 |
| Saudi Arabia | 26.9 | 25.6 | 25.7 | 26.5 | 25.0 |  | 1.8 | 1.2 | 1.2 | 1.8 | 0.8 |
| Tunisia | 37.6 | 35.3 | 36.4 | 34.3 | 32.7 |  | 1.4 | 0.3 | 0.8 | 1.4 | 0.0 |
| UAE | 13.9 | 11.6 | 12.7 | 13.6 | 11.4 |  | 2.5 | 1.4 | 1.9 | 2.5 | 1.0 |
| Yemen | 27.2 | 24.1 | 26.0 | 19.9 | 18.7 |  | 5.9 | 4.4 | 5.3 | 5.9 | 4.1 |

Current values represent the most recent observed smoking prevalence (%). Scenario columns present projected smoking prevalence under hypothetical policy changes derived from regression coefficients (Supplementary Table 2).

Projections are expressed as percentage prevalence of current tobacco smoking among adults, stratified by gender.

For women, no change was applied in the literacy scenario where no statistically significant association was observed between literacy rate and smoking prevalence.
