# Supplementary material for: Tobacco control policies on cancer prevention in the Eastern Mediterranean Region, 2025–2050: A modeling study
Source: PLoS Med. 2026 Apr 24;23(4):e1005032. doi: 10.1371/journal.pmed.1005032 (PMC13108767; doi:10.1371/journal.pmed.1005032)
Supplement: S4 Table — (DOCX) [file pmed.1005032.s004.docx]

**S4 Table:** Projected number and proportion of all incident tobacco-related cancers, for the years 2025 to 2050, that would be attributable to current tobacco smoking in EMR countries.

| Country | N of projected all tobacco-related incident cancers | N of projected cancers attributable to tobacco smoking (95% CI) | PAF of projected cancers related to current tobacco smoking (95% CI) |
| --- | --- | --- | --- |
| Afghanistan | 470,284 | 58,550 (45,605, 73,164) | 12.4 (9.7, 15.6) |
| Bahrain | 33,583 | 7,900 (6,824, 8,976) | 23.5 (20.3, 26.7) |
| Egypt | 3,136,020 | 838,909 (758,695, 942,620) | 26.8 (24.2, 30.1) |
| Iran (Islamic Republic of) | 3,549,157 | 459,695 (370,854, 566,185) | 13.0 (10.4, 16.0) |
| Iraq | 685,665 | 185,244 (164,922, 207,400) | 27.0 (24.1, 30.2) |
| Jordan | 262,559 | 95,843 (87,494, 104,191) | 36.5 (33.3, 39.7) |
| Kuwait | 140,381 | 32,448 (28,293, 36,757) | 23.1 (20.2, 26.2) |
| Lebanon | 152,411 | 58,337 (52,715, 63,960) | 38.3 (34.6, 42.0) |
| Morocco | 1,082,549 | 296,610 (264,587, 333,123) | 27.4 (24.4, 30.8) |
| Oman | 76,455 | 9,754 (7,960, 11,954) | 12.8 (10.4, 15.6) |
| Pakistan | 3,092,670 | 648,419 (538,748, 760,048) | 21.0 (17.4, 24.6) |
| Qatar | 31,792 | 5,383 (4,490, 6,321) | 16.9 (14.1, 19.9) |
| Saudi Arabia | 695,198 | 130,812 (112,063, 150,554) | 18.8 (16.1, 21.7) |
| Tunisia | 412,300 | 141,249 (127,407, 155,850) | 34.3 (30.9, 37.8) |
| United Arab Emirates | 123,933 | 15,649 (12,134, 19,266) | 12.6 (9.8, 15.5) |
| Yemen | 363,076 | 66,126 (55,410, 76,843) | 18.2 (15.3, 21.2) |
| EMR countries | 14,308,033 | 3,050,928 (2,638,201, 3,517,213) | 21.3 (18.4, 24.6) |

This table presents the total projected number of tobacco-related incident cancers, the projected number of cancers attributable to the current prevalence of tobacco smoking, and the corresponding population-attributable fractions (PAFs) with 95% confidence intervals (CIs) for each EMR country.

Projections cover the 25-year period from 2025 to 2050 and are based on cancer incidence estimates from the Global Cancer Observatory (GLOBOCAN), International Agency for Research on Cancer (IARC).

PAF represents the proportion of projected tobacco-related cancers attributable to current tobacco smoking.

EMR = Eastern Mediterranean Region; CI = Confidence Interval.
