# Supplementary material for: Tobacco control policies on cancer prevention in the Eastern Mediterranean Region, 2025–2050: A modeling study
Source: PLoS Med. 2026 Apr 24;23(4):e1005032. doi: 10.1371/journal.pmed.1005032 (PMC13108767; doi:10.1371/journal.pmed.1005032)
Supplement: S5 Table — (DOCX) [file pmed.1005032.s005.docx]

**S5Table.** Relative risks for the associations between current tobacco smoking and associated cancer types and gender

|  | **Men** | **Women** |
| --- | --- | --- |
| **Cancer site** | **RR (95% CI)** | **RR (95% CI)** |
| Lung, bronchus, trachea (C33–C)^1^ | 8.78 (6.13, 12.57) | 7.48 (5.29, 10.60) |
| Larynx (C32) ^2^ | 7.01 (5.56, 8.85) | 7.01 (5.56, 8.85) |
| Esophageal (C15) ^2^ | 2.50 (2.00, 3.13) | 2.03 (1.77, 2.33) |
| Oral Cavity (C00–C13) ^2^ | 3.43 (2.37, 4.94) | 3.43 (2.37, 4.94) |
| Stomach (C16) ^3^ | 1.54 (1.35-1.75) | 1.34 (1.18-1.52) |
| Colorectum (C18–C20, C26.0) ^4^ | 1.19 (1.11, 1.21) | 1.17 (1.09, 1.25) |
| Liver (C22.0, C22.2-C22.4, C22.7, C22.9)^5^ | 1.85 (1.21, 2.83) | 1.49 (1.12, 1.98) |
| Pancreas (C25)^6^ | 1.70 (1.60, 1.90) | 1.90 (1.70, 2.0) |
| Acute myeloid leukemia  (C92.0, C92.4-C92.5, C94.0, C94.2) ^7^ | 1.42 (1.12, 1.81) | 1.28 (1.03, 1.60) |
| Urinary bladder (C67)^8^ | 3.30 (2.9, 3.7) | 3.1 (2.61, 3.58) |
| Kidney, renal pelvis, ureter(C64–C66)^9^ | 1.57 (1.38, 1.77) | 1.27 (1.06, 1.51) |
| Cervix (C53)^10^ | ------ | 1.7 (1.53, 1.88) |
| Pharynx(C14)^2^ | 6.76 (2.86, 16.0) | 6.76 (2.86, 16.0) |

Relative risks (RRs) and 95% confidence intervals (CIs) represent the association between current tobacco smoking and incidence of specific cancer types, stratified by gender. These RRs were used to estimate population-attributable fractions (PAFs) for tobacco-related cancers.

RR= Relative Risk, CI= Confidence Interval

**References**

1 O'Keeffe, L. M. *et al.* Smoking as a risk factor for lung cancer in women and men: a systematic review and meta-analysis. *BMJ Open* **8**, e021611 (2018). <https://doi.org:10.1136/bmjopen-2018-021611>

2 Gandini, S. *et al.* Tobacco smoking and cancer: a meta-analysis. *Int J Cancer* **122**, 155-164 (2008). <https://doi.org:10.1002/ijc.23033>

3 Rota, M. *et al.* Dose-response association between cigarette smoking and gastric cancer risk: a systematic review and meta-analysis. *Gastric Cancer* **27**, 197-209 (2024). <https://doi.org:10.1007/s10120-023-01459-1>

4 Botteri, E. *et al.* Smoking and Colorectal Cancer Risk, Overall and by Molecular Subtypes: A Meta-Analysis. *Am J Gastroenterol* **115**, 1940-1949 (2020). <https://doi.org:10.14309/ajg.0000000000000803>

5 Lee, Y. C. *et al.* Meta-analysis of epidemiologic studies on cigarette smoking and liver cancer. *Int J Epidemiol* **38**, 1497-1511 (2009). <https://doi.org:10.1093/ije/dyp280>

6 Lugo, A. *et al.* Strong excess risk of pancreatic cancer for low frequency and duration of cigarette smoking: A comprehensive review and meta-analysis. *Eur J Cancer* **104**, 117-126 (2018). <https://doi.org:10.1016/j.ejca.2018.09.007>

7 Fircanis, S., Merriam, P., Khan, N. & Castillo, J. J. The relation between cigarette smoking and risk of acute myeloid leukemia: an updated meta-analysis of epidemiological studies. *Am J Hematol* **89**, E125-132 (2014). <https://doi.org:10.1002/ajh.23744>

8 van Osch, F. H., Jochems, S. H., van Schooten, F. J., Bryan, R. T. & Zeegers, M. P. Quantified relations between exposure to tobacco smoking and bladder cancer risk: a meta-analysis of 89 observational studies. *Int J Epidemiol* **45**, 857-870 (2016). <https://doi.org:10.1093/ije/dyw044>

9 Liu, X. *et al.* Dose-response relationships between cigarette smoking and kidney cancer: A systematic review and meta-analysis. *Crit Rev Oncol Hematol* **142**, 86-93 (2019). <https://doi.org:10.1016/j.critrevonc.2019.07.019>

10 Malevolti, M. C. *et al.* Dose-risk relationships between cigarette smoking and cervical cancer: a systematic review and meta-analysis. *Eur J Cancer Prev* **32**, 171-183 (2023). <https://doi.org:10.1097/cej.0000000000000773>
