# Supplementary material for: Tobacco control policies on cancer prevention in the Eastern Mediterranean Region, 2025–2050: A modeling study
Source: PLoS Med. 2026 Apr 24;23(4):e1005032. doi: 10.1371/journal.pmed.1005032 (PMC13108767; doi:10.1371/journal.pmed.1005032)
Supplement: S6 Table — (DOCX) [file pmed.1005032.s006.docx]

**S6 Table:** Projected number and proportion of all tobacco-related cancers and cancers attributable to current tobacco smoking in EMR countries stratified by cancer site (2025-2050)

| Cancer type | N of projected all tobacco-related incident cancers | N of projected cancers attributable to tobacco smoking (95% CI) | PAF of cancers related to current tobacco smoking prevalence (95% CI) |
| --- | --- | --- | --- |
| Lung | 2,163,906 | 1,122,392 (1,023,174, 1,224,182) | 53.6 (48.6, 58.9) |
| Larynx | 520,210 | 272,173 (244,337, 301,319) | 52.3 (47.0, 57.9) |
| Esophagus | 762,931 | 107,875 (82,947, 135,495) | 14.1 (10.9, 17.8) |
| Pharynx | 315,369 | 136,633 (120,722, 152,786) | 43.3 (38.3, 48.4) |
| Oral cavity | 898,091 | 217,764 (175,139, 263,707) | 24.2 (19.5, 29.4) |
| Stomach | 1,688,837 | 106,895 (78,700, 142,787) | 6.3 (4.7, 8.5) |
| Colorectal | 2,058,021 | 58,247 (43,890, 75,230) | 2.8 (2.1, 3.7) |
| Liver | 2,035,614 | 309,911 (264,958, 362,732) | 15.2 (13.0, 17.8) |
| Pancreas | 591,688 | 65,865 (51,984, 82,532) | 11.1 (8.8, 13.9) |
| Leukemia | 798,061 | 49,416 (38,548, 63,012) | 6.2 (4.8, 7.9) |
| Bladder | 1,505,419 | 521,484 (452,422, 597,665) | 34.6 (30.1, 39.7) |
| Kidney | 443,878 | 38,239 (30,588, 47,195) | 8.6 (6.9, 10.6) |
| Cervix | 526,008 | 7,408 (1,271, 17,820) | 1.4 (0.2, 3.4) |
| All-Tobacco related | 14,308,033 | 3,050,928 (2,638,201, 3,517,213) | 21.3 (18.4, 24.6) |

This table presents the total projected number of tobacco-related incident cancers, the projected number of cancers attributable to the current prevalence of tobacco smoking, and the corresponding population-attributable fractions (PAFs) with 95% confidence intervals (CIs) for each investigated cancer site for the EMR.

Projections cover the 25-year period from 2025 to 2050 and are based on cancer incidence estimates from the Global Cancer Observatory (GLOBOCAN), International Agency for Research on Cancer (IARC).

PAF represents the proportion of projected tobacco-related cancers attributable to current tobacco smoking.

EMR = Eastern Mediterranean Region; CI = Confidence Interval.
