# Supplementary material for: Tobacco control policies on cancer prevention in the Eastern Mediterranean Region, 2025–2050: A modeling study
Source: PLoS Med. 2026 Apr 24;23(4):e1005032. doi: 10.1371/journal.pmed.1005032 (PMC13108767; doi:10.1371/journal.pmed.1005032)
Supplement: S7 Table — (DOCX) [file pmed.1005032.s007.docx]

**S7 Table**: Estimated number and proportion of preventable cancer cases attributable to each policy measure for each country stratified by gender and level of Human Development Index (HDI) in the adult population of EMR in 2025-2050

| Both genders |  | | | | |
| --- | --- | --- | --- | --- | --- |
|  | **MPOWER** | |  | **Affordability index** | |
| Country | **PIF (95% CI)** | **N of attributable (95% CI)** |  | **PIF (95% CI)** | **N of attributable (95% CI)** |
| **High HDI** |  | | | | |
| Bahrain | 1.3 (0.9, 1.7) | 441 (308, 573) |  | 0.8 (0.6, 0.9) | 257 (211, 303) |
| Kuwait | 1.7 (1.3, 2.0) | 2,355 (1,866, 2,844) |  | 0.6 (0.5, 0.8) | 905 (739, 1071) |
| Qatar | 1.2 (0.9, 1.5) | 379 (274, 484) |  | 0.8 (0.7, 0.9) | 254 (218, 290) |
| Saudi Arabia | 0.7 (0.4, 1.0) | 4,781 (2,651, 6,910) |  | 0.7 (0.5, 0.8) | 4,529 (3,794, 5,265) |
| United Arab Emirates | 1.8 (1.5, 2.1) | 2,239 (1,839, 2,639) |  | 0.9 (0.8, 1.0) | 1,151 (1,013, 1,289) |
| Oman | 2.2 (1.9, 2.5) | 1,695 (1,460, 1,927) |  | 0.8 (0.7, 0.9) | 592 (514, 668) |
| Total | **1.1 (0.8, 1.4)** | **11,890 (8,397, 15,378)** |  | **0.7 (0.6, 0.8)** | **7,688 (6,489, 8,886)** |
| **Medium HDI** |  | | | | |
| Iran | 0.6 (0.2, 0.9) | 20,708 (8,470, 32,831) |  | 0.9 (0.8, 1.1) | 33,075 (28,578, 37,294) |
| Jordan | 0.3 (0.0, 0.7) | 793 (16, 1,848) |  | 0.4 (0.0, 0.5) | 942 (98, 1,307) |
| Lebanon | 0.8 (0.3, 1.3) | 1,253 (530, 1,975) |  | 0.4 (0.3, 0.6) | 653 (391, 915) |
| Egypt | 0.6 (0.0, 0.9) | 18,696 (1,016, 28,938) |  | 0.4 (0.1, 0.5) | 13,936 (1,616, 17,187) |
| Tunisia | 1.3 (0.8, 1.8) | 5,365 (3,126, 7,343) |  | 0.7 (0.5, 0.8) | 2,779 (1,952, 3,468) |
| Total | **0.6 (0.2, 1.0)** | **46,815 (13,158, 72,935)** |  | **0.7 (0.4, 0.8)** | **51,385 (32,636, 60,170)** |
| **Low HDI** |  | | | | |
| Afghanistan | 2.9 (2.6, 3.3) | 13,674 (12,029, 15,319) |  | 1.0 (0.8, 1.1) | 4,540 (3,947, 5,133) |
| Pakistan | 1.6 (1.1, 2.0) | 48,907 (35,420, 62,394) |  | 0.9 (0.8, 1.1) | 28,603 (23,512, 33,694) |
| Yemen | 1.7 (1.3, 2.0) | 6,068 (4,803, 7,333) |  | 0.6 (0.5, 0.8) | 2,340 (1,830, 2,850) |
| Iraq | 1.1 (0.7, 1.5) | 7,525 (4,651, 10,284) |  | 0.6 (0.5, 0.8) | 4,411 (3,380, 5,373) |
| Morocco | 1.8 (1.3, 2.3) | 19,329 (13,736, 24,388) |  | 0.9 (0.7, 1.1) | 9,734 (7,587, 11,515) |
| Total | **1.7 (1.2, 2.2)** | **95,504 (70,640, 119,719)** |  | **0.9 (0.7, 1.0)** | **49,629 (40,256, 58,565)** |
|  | **Maximizing literacy rate** | | **All policies combined** | | |
| Country | **PIF (95% CI)** | **N of attributable (95% CI)** |  | **PIF (95% CI)** | **N of attributable (95% CI)** |
| **High HDI** |  |  |  |  |  |
| Bahrain | 0.2 (0.0, 1.7) | 51 (1, 577) |  | 1.6 (0.4, 3.3) | 522 (136, 1,105) |
| Kuwait | 0.3 (0.0, 1.6) | 375 (11, 2,206) |  | 1.9 (0.8, 3.4) | 2,639 (1,098, 4,725) |
| Qatar | 0.9 (0.1, 2.1) | 290 (26, 680) |  | 2.2 (0.7, 3.6) | 684 (233, 1,134) |
| Saudi Arabia | 0.1 (0.0, 1.3) | 986 (42, 8,726) |  | 1.0 (0.3, 2.4) | 7,286 (1,945, 16,498) |
| United Arab Emirates | 0.2 (0.0, 1.3) | 216 (9, 1,592) |  | 2.1 (0.8, 3.3) | 2,545 (962, 4,126) |
| Oman | 0.2 (0.0, 1.3) | 158 (8, 1,020) |  | 2.0 (0.7, 3.3) | 1,552 (509, 2,534) |
| Total | **0.2 (0.0, 1.3)** | **2,075 (98, 1,480)** |  | **1.4 (0.4, 2.7)** | **15,228 (4,883, 30,121)** |
| **Medium HDI** |  |  |  |  |  |
| Iran | 1.0 (0.0, 2.1) | 34,564 (1,654, 76,081) |  | 1.9 (0.5, 3.2) | 66,729 (19,218, 112,774) |
| Jordan | 0.1 (0.0, 1.7) | 182 (18, 4,375) |  | 0.5 (0.0, 2.3) | 1,360 (76, 6,073) |
| Lebanon | 0.5 (0.0, 2.0) | 782 (35, 2,986) |  | 1.3 (0.3, 3.0) | 2,001 (423, 4,642) |
| Egypt | 1.4 (0.0, 2.6) | 44,254 (512, 81,066) |  | 1.8 (0.0, 3.2) | 55,746 (917, 98,820) |
| Tunisia | 1.2 (0.1, 3.1) | 4,787 (582, 12,808) |  | 2.4 (0.8, 4.6) | 9,803 (3,285, 19,068) |
| Total | **1.1 (0.0, 2.4)** | **84,569 (2,801, 177,317)** |  | **1.8 (0.3, 3.2)** | **135,638 (23,919, 241,378)** |
| **Low HDI** |  |  |  |  |  |
| Afghanistan | 9.5 (8.4, 10.6) | 44,656 (39,598, 49,713) |  | 10.9 (9.7, 12.2) | 51,398 (45,398, 57,398) |
| Pakistan | 4.6 (3.2, 6.0) | 143,125 (100,108, 186,143) |  | 5.7 (4.0, 7.3) | 175,302 (124,391, 226,213) |
| Iraq | 0.7 (0.1, 2.3) | 4,941 (378, 15,605) |  | 1.9 (0.8, 3.6) | 12,857 (5,179, 24,919) |
| Morocco | 2.1 (0.4, 4.0) | 22,948 (4,077, 43,698) |  | 3.5 (1.3, 5.7) | 38,278 (13,941, 62,113) |
| Yemen | 2.4 (1.5, 3.4) | 8,794 (5,360, 12,228) |  | 3.7 (2.6, 4.9) | 13,591 (9,277, 17,905) |
| Total | **3.9 (2.6, 5.4)** | **224,463 (149,521, 307,386)** |  | **5.1 (3.2, 6.8)** | **291,425 (198,186, 388,546)** |

| **Men** |  | | | | |
| --- | --- | --- | --- | --- | --- |
|  | **MPOWER** | |  | **Affordability index** | |
| Country | **PIF (95% CI)** | **N of attributable (95% CI)** |  | **PIF (95% CI)** | **N of attributable (95% CI)** |
| **High HDI** |  | | | | |
| Bahrain | 1.4 (1.0, 1.9) | 336 (229, 442) |  | 0.8 (0.7, 1.0) | 195 (160, 230) |
| Kuwait | 1.5 (1.1, 1.9) | 1,539 (1,155, 1,923) |  | 0.6 (0.5, 0.7) | 591 (468, 714) |
| Qatar | 1.3 (0.9, 1.7) | 283 (200, 366) |  | 0.9 (0.7, 1.0) | 189 (162, 215) |
| Saudi Arabia | 0.7 (0.3, 1.0) | 3,434 (1,735, 5,134) |  | 0.6 (0.5, 0.8) | 3,238 (2,688, 3,789) |
| United Arab Emirates | 2.1 (1.7, 2.5) | 1,632 (1,343, 1,922) |  | 1.1 (1.0, 1.2) | 834 (740, 927) |
| Oman | 2.9 (2.6, 3.3) | 1,624 (1,437, 1,811) |  | 0.9 (0.8, 1.0) | 521 (463, 579) |
| Total | 1.1 (0.8, 1.5) | **8,848 (6,099, 11,597)** |  | **0.7 (0.6, 0.8)** | **5,567 (4,681, 6,453)** |
| **Medium HDI** |  | | | | |
| Iran | 0.3 (0.0, 0.8) | 543 (18, 1,397) |  | 1.0 (0.9, 1.1) | 22,122 (19,548, 24,696) |
| Jordan | 0.5 (0.0, 3.0) | 903 (18, 5,392) |  | 0.4 (0.0, 0.5) | 642 (20, 923) |
| Lebanon | 0.9 (0.4, 1.4) | 828 (395, 1,262) |  | 0.5 (0.3, 0.6) | 429 (284, 573) |
| Egypt | 1.2 (0.6, 1.7) | 3,323 (1,682, 4,964) |  | 0.6 (0.4, 0.8) | 1,714 (1,164, 2,264) |
| Tunisia | 0.7 (0.0, 1.1) | 14,457 (200, 22,151) |  | 0.5 (0.0, 0.6) | 9,697 (228, 11,912) |
| Total | **0.7 (0.1, 1.3)** | **20,054 (2,312, 35,167)** |  | **0.7 (0.4, 0.9)** | **34,604 (21,244, 40,369)** |
| **Low HDI** |  | | | | |
| Afghanistan | 3.7 (3.3, 4.1) | 9,375 (8,280, 10,469) |  | 1.2 (1.0, 1.3) | 2,985 (2,649, 3,322) |
| Pakistan | 1.7 (1.2, 2.3) | 31,660 (22,122, 41,199) |  | 1.0 (0.8, 1.2) | 18,386 (15,340, 21,432) |
| Yemen | 1.8 (1.4, 2.2) | 3,526 (2,751, 4,301) |  | 0.7 (0.6, 0.8) | 1,349 (1,106, 1,593) |
| Iraq | 1.0 (0.5, 1.5) | 4,308 (2,176, 6,440) |  | 0.6 (0.4, 0.7) | 2,513 (1,814, 3,211) |
| Morocco | 2.1 (1.5, 2.7) | 14,888 (10,722, 19,053) |  | 1.0 (0.8, 1.2) | 6,811 (5,393, 8,229) |
| Total | **1.9 (1.3, 2.4)** | **63,757 (46,051, 81,462)** |  | **0.9 (0.8, 1.1)** | **32,045 (26,303, 37,786)** |
|  | **Maximizing literacy rate** | | **All policies combined** | | |
| Country | **PIF (95% CI)** | **N of attributable (95% CI)** |  | **PIF (95% CI)** | **N of attributable (95% CI)** |
| **High HDI** |  |  |  |  |  |
| Bahrain | 0.2 (0.0, 2.4) | 51 (1, 577) |  | 1.6 (0.1, 3.9) | 379 (22, 932) |
| Kuwait | 0.4 (0.0, 2.2) | 375 (11, 2,206) |  | 1.6 (0.2, 3.6) | 1,654 (231, 3,622) |
| Qatar | 1.3 (0.1, 3.1) | 290 (26, 680) |  | 2.5 (0.5, 4.4) | 546 (120, 971) |
| Saudi Arabia | 0.2 (0.0, 1.7) | 986 (42, 8,726) |  | 1.0 (0.0, 2.8) | 5,062 (203, 13,792) |
| United Arab Emirates | 0.3 (0.0, 2.0) | 216 (9, 1592) |  | 2.2 (0.4, 4.1) | 1,748 (288, 3,207) |
| Oman | 0.3 (0.0, 1.8) | 158 (8, 1020) |  | 2.7 (0.9, 4.4) | 1,481 (487, 2,414) |
| Total | **0.3 (0.0, 1.9)** | **2,075 (98, 14,801)** |  | **1.4 (0.2, 3.2)** | **10,871 (1,351, 24,939)** |
| **Medium HDI** |  |  |  |  |  |
| Iran | 1.6 (0.1, 3.5) | 34,564 (1,654, 76,081) |  | 2.4 (0.4, 4.3) | 52,048 (8,894, 94,060) |
| Jordan | 0.1 (0.0, 2.5) | 182 (18, 4,375) |  | 0.5 (0.0, 3.0) | 903 (18, 5,392) |
| Lebanon | 0.9 (0.0, 3.4) | 782 (35, 2,986) |  | 1.6 (0.2, 4.2) | 1448 (190, 3,769) |
| Egypt | 1.7 (0.2, 4.5) | 4,787 (582, 12,808) |  | 2.5 (0.5, 5.6) | 7,230 (1,419, 16,117) |
| Tunisia | 2.2 (0.0, 4.1) | 44,254 (512, 81,066) |  | 2.6 (0.0, 4.6) | 51,507 (200, 91,745) |
| Total | **1.8 (0.1, 3.7)** | **84,569 (2,801, 177,317)** |  | **2.4 (0.2, 4.4)** | **113,136 (10,721, 211,084)** |
| **Low HDI** |  |  |  |  |  |
| Afghanistan | 17.6 (15.6, 19.6) | 44,656 (39,598, 49,713) |  | 18.6 (16.5, 20.7) | 47,098 (41,706, 52,490) |
| Pakistan | 7.8 (5.5, 10.2) | 143,125 (100,108, 186,143) |  | 8.3 (5.8, 10.9) | 151,806 (105,272, 198,340) |
| Yemen | 4.5 (2.8, 6.3) | 8,794 (5,360, 12,228) |  | 5.4 (3.5, 7.3) | 10,536 (6,770, 14,302) |
| Iraq | 1.1 (0.1, 3.5) | 4,941 (378, 15,605) |  | 1.9 (0.4, 4.5) | 8,458 (1,636, 19,825) |
| Morocco | 3.2 (0.6, 6.1) | 22,948 (4,077, 43,698) |  | 4.7 (1.5, 8.0) | 33,837 (11,000, 56,674) |
| Total | **6.5 (4.4, 9.0)** | **224,463 (149,521, 307,386)** |  | **7.3 (4.9, 10.0)** | **251,735 (166,383, 341,632)** |

| **Women** |  | | | | |
| --- | --- | --- | --- | --- | --- |
|  | **MPOWER** | |  | **Affordability index** | |
| Country | **PIF (95% CI)** | **N of attributable (95% CI)** |  | **PIF (95% CI)** | **N of attributable (95% CI)** |
| **High HDI** |  | | | | |
| Bahrain | 1.1 (0.8, 1.3) | 105 (79, 131) |  | 0.6 (0.5, 0.7) | 62 (51, 73) |
| Kuwait | 2.0 (1.8, 2.3) | 816 (711, 922) |  | 0.8 (0.7, 0.9) | 314 (271, 357) |
| Qatar | 1.0 (0.7, 1.2) | 96 (74, 119) |  | 0.7 (0.6, 0.8) | 65 (55, 75) |
| Saudi Arabia | 0.7 (0.5, 0.9) | 1,346 (916, 1,776) |  | 0.7 (0.6, 0.8) | 1,291 (1,106, 1,476) |
| United Arab Emirates | 1.3 (1.1, 1.6) | 607 (496, 718) |  | 0.7 (0.6, 0.8) | 317 (273, 362) |
| Oman | 0.3 (0.1, 0.5) | 71 (23, 116) |  | 0.3 (0.2, 0.4) | 71 (51, 90) |
| Total | **0.9 (0.7, 1.2)** | **3,042 (2,299, 3,781)** |  | **0.7 (0.6, 0.8)** | **2,122 (1,808, 2,433)** |
| **Medium HDI** |  | | | | |
| Iran | 0.5 (0.2, 0.8) | 6,777 (3,002, 10,435) |  | 0.8 (0.7, 0.9) | 10,953 (9,031, 12,597) |
| Jordan | 0.3 (0.0, 0.5) | 250 (1, 451) |  | 0.4 (0.1, 0.5) | 301 (78, 384) |
| Lebanon | 0.7 (0.2, 1.1) | 424 (136, 713) |  | 0.4 (0.2, 0.5) | 224 (107, 342) |
| Egypt | 1.6 (1.1, 1.9) | 2,042 (1,445, 2,379) |  | 0.8 (0.6, 0.9) | 1,065 (789, 1,204) |
| Tunisia | 0.4 (0.1, 0.6) | 4,239 (816, 6,787) |  | 0.4 (0.1, 0.5) | 4,239 (1,388, 5,275) |
| Total | **0.5 (0.2, 0.8)** | **13,733 (5,400, 20,766)** |  | **0.6 (0.4, 0.7)** | **16,782 (11,392, 19,801)** |
| **Low HDI** |  | | | | |
| Afghanistan | 2.0 (1.7, 2.2) | 4,299 (3,749, 4,850) |  | 0.7 (0.6, 0.8) | 1,555 (1,299, 1,811) |
| Pakistan | 1.4 (1.1, 1.7) | 17,247 (13,299, 21,195) |  | 0.8 (0.6, 1.0) | 10,217 (8,172, 12,262) |
| Yemen | 1.5 (1.2, 1.8) | 2,542 (2,052, 3,032) |  | 0.6 (0.4, 0.7) | 990 (723, 1,257) |
| Iraq | 1.3 (1.0, 1.6) | 3,217 (2,475, 3,845) |  | 0.8 (0.6, 0.9) | 1,899 (1,565, 2,162) |
| Morocco | 1.2 (0.8, 1.4) | 4,442 (3,014, 5,335) |  | 0.8 (0.6, 0.9) | 2,923 (2,194, 3,286) |
| Total | **1.4 (1.1, 1.7)** | **31,747 (24,589, 38,257)** |  | **0.8 (0.6, 0.9)** | **17,584 (13,953, 20,779)** |
|  | **Maximizing literacy rate** | | **All policies combined** | | |
| Country | **PIF (95% CI)** | **N of attributable (95% CI)** |  | **PIF (95% CI)** | **N of attributable (95% CI)** |
| **Very high HDI** | ------ | ---- |  | 1.4 (1.2, 1.7) | 143 (114, 172) |
| Bahrain | ------ | ---- |  | 2.5 (2.2, 2.8) | 985 (867, 1103) |
| Kuwait | ------ | ---- |  | 1.4 (1.1, 1.6) | 138 (113, 163) |
| Qatar | ------ | ---- |  | 1.1 (0.9, 1.4) | 2,224 (1,742, 2,705) |
| Saudi Arabia | ------ | ---- |  | 1.7 (1.5, 2.0) | 796 (674, 919) |
| United Arab Emirates | ------ | ---- |  | 0.3 (0.1, 0.6) | 71 (22, 120) |
| Oman | ------ | ---- |  | 1.3 (1.1, 1.6) | 4,357 (3,532, 5,182) |
| Total | ------ | ---- |  | **1.4 (1.2, 1.7)** | **143 (114, 172)** |
| **Medium HDI** |  |  |  |  |  |
| Iran | ------ | ---- |  | 1.1 (0.8, 1.4) | 14,680 (10,324, 18,714) |
| Jordan | ------ | ---- |  | 0.5 (0.1, 0.8) | 457 (58, 681) |
| Lebanon | ------ | ---- |  | 0.9 (0.4, 1.4) | 553 (233, 873) |
| Egypt | ------ | ---- |  | 2.0 (1.5, 2.3) | 2,573 (1,865, 2,951) |
| Tunisia | ------ | ---- |  | 0.4 (0.1, 0.6) | 4,239 (717, 7,075) |
| Total | ------ | ---- |  | **0.8 (0.5, 1.1)** | **22,502 (13,197, 30,294)** |
| **Low HDI** |  |  |  |  |  |
| Afghanistan | ------ | ---- |  | 2.0 (1.7, 2.3) | 4,299 (3,692, 4,907) |
| Pakistan | ------ | ---- |  | 1.9 (1.5, 2.2) | 23,496 (19,119, 27,872) |
| Yemen | ------ | ---- |  | 1.8 (1.5, 2.1) | 3,055 (2,508, 3,603) |
| Iraq | ------ | ---- |  | 1.8 (1.4, 2.1) | 4,398 (3,543, 5,093) |
| Morocco | ------ | ---- |  | 1.2 (0.8, 1.5) | 4,442 (2,941, 5,439) |
| Total | ------ | ---- |  | **1.8 (1.4, 2.1)** | **39,690 (31,803, 46,914)** |

This table presents the projected number and proportion of preventable tobacco-related cancer cases under different policy scenarios, including improvement in MPOWER implementation score, reduction in tobacco affordability, maximization of literacy rate, and all policies combined. Estimates are stratified by country, gender, and HDI category (high, medium, and low HDI). *Preventable fractions (PIFs) represent the proportion of projected tobacco-related cancers that could be avoided under each policy scenario compared with the baseline scenario (current policy levels). The number of preventable cases was calculated by applying PIFs to projected cancer incidence for the period 2025–2050.

PIF = Potential Impact Fraction; HDI = Human Development Index; EMR = Eastern Mediterranean Region; CI = Confidence Interval
