# Supplementary material for: Tobacco control policies on cancer prevention in the Eastern Mediterranean Region, 2025–2050: A modeling study
Source: PLoS Med. 2026 Apr 24;23(4):e1005032. doi: 10.1371/journal.pmed.1005032 (PMC13108767; doi:10.1371/journal.pmed.1005032)
Supplement: S8 Table — (DOCX) [file pmed.1005032.s008.docx]

**S8 Table:** Number of projected preventable cancers by 2050, that could be achieved though highest MPOWER implementation, a 10-unit increase in tobacco affordability index, maximizing literacy rate, and combined implementations of all policies in EMR countries assuming cancer incidence is 10% higher than GLOBOCAN estimates

| **Both genders** | **Preventable cancer by highest MPOWER** | | **Preventable cancer by a 10-unit increases in tobacco affordability index** | |
| --- | --- | --- | --- | --- |
| Country | PIF (95% CI) | N of cancer (95% CI) | PIF (95% CI) | N of cancer (95% CI) |
| Afghanistan | 2.9 (2.6, 3.3) | 15,000 (13,000, 17,000) | 1.0 (0.8, 1.1) | 5,000 (4,000, 6,000) |
| Bahrain | 1.3 (0.9, 1.7) | 485 (339, 630) | 0.8 (0.6, 0.9) | 283 (232, 333) |
| Egypt | 0.6 (0.0, 0.9) | 21,000 (1,000, 32,000) | 0.4 (0.1, 0.5) | 15,000 (2,000, 19,000) |
| Iran | 0.6 (0.2, 0.9) | 23,000 (9,000, 36,000) | 0.9 (0.8, 1.1) | 36,000 (31,000, 41,000) |
| Iraq | 1.1 (0.7, 1.5) | 8,000 (5,000, 11,000) | 0.6 (0.5, 0.8) | 5,000 (4,000, 6,000) |
| Jordan | 0.3 (0.0, 0.7) | 1,000 (0, 2,000) | 0.4 (0.0, 0.5) | 1,000 (108, 1,000) |
| Kuwait | 1.7 (1.3, 2.0) | 3,000 (2,000, 3,000) | 0.6 (0.5, 0.8) | 996 (813, 1,000) |
| Lebanon | 0.8 (0.3, 1.3) | 1,000 (1,000, 2,000) | 0.4 (0.3, 0.6) | 718 (430, 1,000) |
| Morocco | 1.8 (1.3, 2.3) | 21,000 (15,000, 27,000) | 0.9 (0.7, 1.1) | 11,000 (8,000, 13,000) |
| Oman | 2.2 (1.9, 2.5) | 2,000 (2,000, 2,000) | 0.8 (0.7, 0.9) | 651 (565, 735) |
| Pakistan | 1.6 (1.1, 2.0) | 54,000 (39,000, 69,000) | 0.9 (0.8, 1.1) | 31,000 (26,000, 37,000) |
| Qatar | 1.2 (0.9, 1.5) | 417 (301, 532) | 0.8 (0.7, 0.9) | 279 (240, 319) |
| Saudi Arabia | 0.7 (0.4, 1.0) | 5,000 (3,000, 8,000) | 0.7 (0.5, 0.8) | 5,000 (4,000, 6,000) |
| Tunisia | 1.3 (0.8, 1.8) | 6,000 (3,000, 8,000) | 0.7 (0.5, 0.8) | 3,000 (2,000, 4,000) |
| United Arab Emirates | 1.8 (1.5, 2.1) | 2,000 (2,000, 3,000) | 0.9 (0.8, 1.0) | 1,000 (1,000, 1,000) |
| Yemen | 1.7 (1.3, 2.0) | 7,000 (5,000, 8,000) | 0.6 (0.5, 0.8) | 3,000 (2,000, 3,000) |
| EMRO | 1.1 (0.6, 1.5) | 170,000 (101,000, 229,000) | 0.8 (0.6, 0.9) | 120,000 (87,000, 140,000) |
|  | **Preventable cancer from maximizing literacy rate** | | **Preventable cancers from combined implementation of all policies** | |
| Country | PIF (95% CI) | N of cancer (95% CI) | PIF (95% CI) | N of cancer (95% CI) |
| Afghanistan | 9.5 (8.4, 10.6) | 49,000 (44,000, 55,000) | 10.9 (9.7, 12.2) | 57,000 (50,000, 63,000) |
| Bahrain | 0.2 (0.0, 1.7) | 56 (1, 635) | 1.6 (0.4, 3.3) | 574 (150, 1,000) |
| Egypt | 1.4 (0.0, 2.6) | 49,000 (563, 89,000) | 1.8 (0.0, 3.2) | 61,000 (1,000, 109,000) |
| Iran | 1.0 (0.0, 2.1) | 38,000 (1,819, 84,000) | 1.9 (0.5, 3.2) | 73,000 (21,000, 124,000) |
| Iraq | 0.7 (0.1, 2.3) | 5,000 (416, 17,000) | 1.9 (0.8, 3.6) | 14,000 (6,000, 27,000) |
| Jordan | 0.1 (0.0, 1.7) | 200 (20, 4,813) | 0.5 (0.0, 2.3) | 1,000 (84, 7,000) |
| Kuwait | 0.3 (0.0, 1.6) | 413 (12, 2,427) | 1.9 (0.8, 3.4) | 3,000 (1,000, 5,000) |
| Lebanon | 0.5 (0.0, 2.0) | 860 (39, 3,285) | 1.3 (0.3, 3.0) | 2,000 (465, 5,000) |
| Morocco | 2.1 (0.4, 4.0) | 25,000 (4,485, 48,000) | 3.5 (1.3, 5.7) | 42,000 (15,000, 68,000) |
| Oman | 0.2 (0.0, 1.3) | 174 (9, 1,122) | 2.0 (0.7, 3.3) | 2,000 (560, 3,000) |
| Pakistan | 4.6 (3.2, 6.0) | 157,000 (110,000, 205,000) | 5.7 (4.0, 7.3) | 193,000 (137,000, 249,000) |
| Qatar | 0.9 (0.1, 2.1) | 319 (29, 748) | 2.2 (0.7, 3.6) | 752 (256, 1,000) |
| Saudi Arabia | 0.1 (0.0, 1.3) | 1,085 (46, 9,599) | 1.0 (0.3, 2.4) | 8,000 (2,000, 18,000) |
| Tunisia | 1.2 (0.1, 3.1) | 5,000 (640, 14,000) | 2.4 (0.8, 4.6) | 11,000 (4,000, 21,000) |
| United Arab Emirates | 0.2 (0.0, 1.3) | 238 (10, 1,751) | 2.1 (0.8, 3.3) | 3,000 (1,000, 5,000) |
| Yemen | 2.4 (1.5, 3.4) | 10,000 (6,000, 13,000) | 3.7 (2.6, 4.9) | 15,000 (10,000, 20,000) |
| EMRO | 2.2 (1.1, 3.5) | 342,000 (168,000, 549,000) | 3.1 (1.6, 4.6) | 487,000 (250,000, 726,000) |

PIF = Potential Impact Fraction; EMR = Eastern Mediterranean Region; CI = Confidence Interval.

This table presents the estimated number and proportion of preventable tobacco-related cancer cases under alternative tobacco control policy scenarios. Estimates were calculated under the assumption that total cancer incidence over the next 25 years (2025–2050) will be 10% higher than the GLOBOCAN projections.

Results are presented at the country level for both genders combined. The 10% increase represents a sensitivity analysis scenario to account for potential underestimation in baseline projections.
