# Supplementary material for: Tobacco control policies on cancer prevention in the Eastern Mediterranean Region, 2025–2050: A modeling study
Source: PLoS Med. 2026 Apr 24;23(4):e1005032. doi: 10.1371/journal.pmed.1005032 (PMC13108767; doi:10.1371/journal.pmed.1005032)
Supplement: S9 Table — (DOCX) [file pmed.1005032.s009.docx]

**S9 Table:** Number of avoidable cancers due to highest MPOWER, a 10-unit increase in affordability index, maximizing level of literacy, and all combined policies in EMR countries (2025-2050) stratified by cancer site assuming cancer incidence is 10% higher than GLOBOCAN estimates

| **Both genders** | **Preventable cancer by highest MPOWER** | | **Preventable cancer by** a 10-unit **increases in tobacco affordability index** | |
| --- | --- | --- | --- | --- |
|  | PIF (95% CI) | N of cancer (95% CI) | PIF (95% CI) | N of cancer (95% CI) |
| Lung | 2.2 (1.3, 3.1) | 52,000 (31,000, 74,000) | 1.6 (1.2, 1.9) | 38,000 (29,000, 46,000) |
| Larynx | 2.1 (1.1, 3.0) | 12,000 (7,000, 17,000) | 1.5 (1.2, 1.9) | 9,000 (7,000, 11,000) |
| Esophagus | 1.2 (0.6, 1.8) | 10,000 (5,000, 15,000) | 0.8 (0.5, 1.1) | 7,000 (4,000, 9,000) |
| Pharynx | 2.9 (1.8, 3.7) | 10,000 (6,000, 13,000) | 1.7 (1.1, 2.1) | 6,000 (4,000, 7,000) |
| Oral cavity | 2.1 (1.6, 2.6) | 21,000 (15,000, 26,000) | 1.3 (1.0, 1.4) | 12,000 (10,000, 14,000) |
| Stomach | 0.5 (0.3, 0.6) | 9,000 (6,000, 11,000) | 0.4 (0.3, 0.4) | 7,000 (6,000, 8,000) |
| Colorectal | 0.2 (0.2, 0.3) | 5,000 (3,000, 6,000) | 0.2 (0.1, 0.2) | 4,000 (3,000, 4,000) |
| Liver | 0.6 (0.3, 0.8) | 14,000 (6,000, 17,000) | 0.4 (0.2, 0.5) | 10,000 (5,000, 11,000) |
| Pancreas | 0.7 (0.4, 0.9) | 5,000 (3,000, 6,000) | 0.5 (0.4, 0.6) | 4,000 (3,000, 4,000) |
| Leukemia | 0.4 (0.2, 0.5) | 4,000 (2,000, 4,000) | 0.3 (0.2, 0.3) | 3,000 (2,000, 3,000) |
| Bladder | 1.3 (0.7, 1.9) | 22,000 (12,000, 31,000) | 1.0 (0.7, 1.2) | 16,000 (11,000, 19,000) |
| Kidney | 0.5 (0.4, 0.6) | 3,000 (2,000, 3,000) | 0.4 (0.3, 0.4) | 2,000 (1,000, 2,000) |
| Cervix | 0.6 (0.5, 0.8) | 4,000 (3,000, 4,000) | 0.6 (0.5, 0.8) | 4,000 (3,000, 4,000) |
| All-Tobacco related | 1.1 (0.6, 1.5) | 170,000 (101,000, 229,000) | 0.8 (0.6, 0.9) | 120,000 (87,000, 140,000) |
|  | **Preventable cancer from maximizing literacy rate** | | **Preventable cancers from combined implementation of all policies** | |
|  | PIF (95% CI) | N of cancer (95% CI) | PIF (95% CI) | N of cancer (95% CI) |
| Lung | 3.8 (1.5, 7.5) | 91,000 (37,000, 179,000) | 6.0 (2.6, 10.3) | 142,000 (63,000, 244,000) |
| Larynx | 5.0 (2.3, 8.8) | 29,000 (13,000, 50,000) | 6.8 (3.2, 11.1) | 39,000 (18,000, 63,000) |
| Esophagus | 2.8 (1.5, 4.2) | 23,000 (13,000, 35,000) | 3.7 (2.5, 4.9) | 31,000 (21,000, 41,000) |
| Pharynx | 5.4 (5.2, 5.7) | 19,000 (18,000, 20,000) | 8.0 (4.6, 11.4) | 28,000 (16,000, 40,000) |
| Oral cavity | 5.4 (3.8, 7.0) | 53,000 (37,000, 69,000) | 6.8 (5.0, 8.6) | 67,000 (50,000, 85,000) |
| Stomach | 1.0 (0.5, 1.5) | 18,000 (9,000, 27,000) | 1.4 (0.8, 1.8) | 25,000 (16,000, 34,000) |
| Colorectal | 0.4 (0.2, 0.5) | 8,000 (4,000, 12,000) | 0.5 (0.3, 0.7) | 12,000 (8,000, 16,000) |
| Liver | 1.5 (0.5, 2.0) | 33,000 (11,000, 44,000) | 1.9 (0.5, 2.6) | 43,000 (12,000, 58,000) |
| Pancreas | 1.1 (0.3, 1.9) | 7,000 (2,000, 12,000) | 1.8 (0.9, 2.4) | 11,000 (6,000, 16,000) |
| Leukemia | 0.7 (0.3, 1.1) | 6,000 (3,000, 9,000) | 1.1 (0.6, 1.4) | 9,000 (5,000, 12,000) |
| Bladder | 3.0 (1.1, 5.1) | 49,000 (18,000, 84,000) | 4.0 (1.7, 6.1) | 66,000 (28,000, 102,000) |
| Kidney | 1.1 (0.7, 1.5) | 6,000 (3,000, 7,000) | 1.5 (0.9, 2.0) | 7,000 (4,000, 10,000) |
| Cervix | 0.0 (0.0, 0.0) | 0 (0, 0) | 0.8 (0.6, 0.9) | 4,000 (4,000, 5,000) |
| All-Tobacco related | 2.2 (1.1, 3.5) | 342,000 (168,000, 549,000) | 3.1 (1.6, 4.6) | 487,000 (250,000, 726,000) |

PIF = Potential Impact Fraction; EMR = Eastern Mediterranean Region; CI = Confidence Interval.

This table presents the estimated number and proportion of preventable tobacco-related cancer cases under alternative tobacco control policy scenarios. Estimates were calculated under the assumption that total cancer incidence over the next 25 years (2025–2050) will be 10% higher than the GLOBOCAN projections.

Results are presented by cancer site for both genders combined. The 10% increase represents a sensitivity analysis scenario to account for potential underestimation in baseline projections.
