# Supplementary material for: Tobacco control policies on cancer prevention in the Eastern Mediterranean Region, 2025–2050: A modeling study
Source: PLoS Med. 2026 Apr 24;23(4):e1005032. doi: 10.1371/journal.pmed.1005032 (PMC13108767; doi:10.1371/journal.pmed.1005032)
Supplement: S10 Table — (DOCX) [file pmed.1005032.s010.docx]

**S10 Table:** Number of projected preventable cancers by 2050, that could be achieved though highest MPOWER implementation, a 10-unit increase in tobacco affordability index, maximizing literacy rate, and combined implementations of all policies in EMR countries assuming cancer incidence is 10% lower than GLOBOCAN estimates

| **Both genders** | **Preventable cancer by highest MPOWER** | | **Preventable cancer by a 10-unit increases in tobacco affordability index** | |
| --- | --- | --- | --- | --- |
| Country | PIF (95% CI) | N of cancer (95% CI) | PIF (95% CI) | N of cancer (95% CI) |
| Afghanistan | 2.9 (2.6, 3.3) | 12,000 (11,000, 14,000) | 1.0 (0.8, 1.1) | 4,000 (4,000, 5,000) |
| Bahrain | 1.3 (0.9, 1.7) | 397 (277, 516) | 0.8 (0.6, 0.9) | 231 (190, 273) |
| Egypt | 0.6 (0.0, 0.9) | 17,000 (914, 26,000) | 0.4 (0.1, 0.5) | 13,000 (1,000, 15,000) |
| Iran | 0.6 (0.2, 0.9) | 19,000 (8,000, 30,000) | 0.9 (0.8, 1.1) | 30,000 (26,000, 34,000) |
| Iraq | 1.1 (0.7, 1.5) | 7,000 (4,000, 9,000) | 0.6 (0.5, 0.8) | 4,000 (3,000, 5,000) |
| Jordan | 0.3 (0.0, 0.7) | 714 (14, 2,000) | 0.4 (0.0, 0.5) | 848 (88, 1,176) |
| Kuwait | 1.7 (1.3, 2.0) | 2,000 (2,000, 3,000) | 0.6 (0.5, 0.8) | 815 (665, 964) |
| Lebanon | 0.8 (0.3, 1.3) | 1,000 (477, 2,000) | 0.4 (0.3, 0.6) | 588 (352, 824) |
| Morocco | 1.8 (1.3, 2.3) | 17,000 (12,000, 22,000) | 0.9 (0.7, 1.1) | 9,000 (7,000, 10,000) |
| Oman | 2.2 (1.9, 2.5) | 2,000 (1,000, 2,000) | 0.8 (0.7, 0.9) | 533 (463, 601) |
| Pakistan | 1.6 (1.1, 2.0) | 44,000 (32,000, 56,000) | 0.9 (0.8, 1.1) | 26,000 (21,000, 30,000) |
| Qatar | 1.2 (0.9, 1.5) | 341 (247, 436) | 0.8 (0.7, 0.9) | 229 (196, 261) |
| Saudi Arabia | 0.7 (0.4, 1.0) | 4,000 (2,000, 6,000) | 0.7 (0.5, 0.8) | 4,000 (3,000, 5,000) |
| Tunisia | 1.3 (0.8, 1.8) | 5,000 (3,000, 7,000) | 0.7 (0.5, 0.8) | 3,000 (2,000, 3,000) |
| United Arab Emirates | 1.8 (1.5, 2.1) | 2,000 (2,000, 2,000) | 0.9 (0.8, 1.0) | 1,000 (1,000, 1,000) |
| Yemen | 1.7 (1.3, 2.0) | 5,000 (4,000, 7,000) | 0.6 (0.5, 0.8) | 2,000 (2,000, 3,000) |
| EMRO | 1.1 (0.6, 1.5) | 139,000 (83,000, 187,000) | 0.8 (0.6, 0.9) | 98,000 (71,000, 115,000) |
|  | **Preventable cancer from maximizing literacy rate** | | **Preventable cancers from combined implementation of all policies** | |
| Country | PIF (95% CI) | N of cancer (95% CI) | PIF (95% CI) | N of cancer (95% CI) |
| Afghanistan | 9.5 (8.4, 10.6) | 40,000 (36,000, 45,000) | 10.9 (9.7, 12.2) | 46,000 (41,000, 52,000) |
| Bahrain | 0.2 (0.0, 1.7) | 46 (1, 519) | 1.6 (0.4, 3.3) | 470 (122, 995) |
| Egypt | 1.4 (0.0, 2.6) | 40,000 (461, 73,000) | 1.8 (0.0, 3.2) | 50,000 (825, 89,000) |
| Iran | 1.0 (0.0, 2.1) | 31,000 (1,000, 68,000) | 1.9 (0.5, 3.2) | 60,000 (17,000, 102,000) |
| Iraq | 0.7 (0.1, 2.3) | 4,000 (340, 14,000) | 1.9 (0.8, 3.6) | 12,000 (5,000, 22,000) |
| Jordan | 0.1 (0.0, 1.7) | 164 (16, 3,938) | 0.5 (0.0, 2.3) | 1,000 (68, 5,000) |
| Kuwait | 0.3 (0.0, 1.6) | 338 (10, 1,985) | 1.9 (0.8, 3.4) | 2,000 (1,000, 4,000) |
| Lebanon | 0.5 (0.0, 2.0) | 704 (32, 2,687) | 1.3 (0.3, 3.0) | 2,000 (381, 4,000) |
| Morocco | 2.1 (0.4, 4.0) | 21,000 (4,000, 39,000) | 3.5 (1.3, 5.7) | 34,000 (13,000, 56,000) |
| Oman | 0.2 (0.0, 1.3) | 142 (7, 918) | 2.0 (0.7, 3.3) | 1,000 (458, 2,000) |
| Pakistan | 4.6 (3.2, 6.0) | 129,000 (90,000, 168,000) | 5.7 (4.0, 7.3) | 158,000 (112,000, 204,000) |
| Qatar | 0.9 (0.1, 2.1) | 261 (23, 612) | 2.2 (0.7, 3.6) | 616 (210, 1,021) |
| Saudi Arabia | 0.1 (0.0, 1.3) | 887 (38, 7,853) | 1.0 (0.3, 2.4) | 7,000 (2,000, 15,000) |
| Tunisia | 1.2 (0.1, 3.1) | 4,000 (524, 12,000) | 2.4 (0.8, 4.6) | 9,000 (3,000, 17,000) |
| United Arab Emirates | 0.2 (0.0, 1.3) | 194 (8, 1,433) | 2.1 (0.8, 3.3) | 2,000 (866, 4,000) |
| Yemen | 2.4 (1.5, 3.4) | 8,000 (5,000, 11,000) | 3.7 (2.6, 4.9) | 12,000 (8,000, 16,000) |
| EMRO | 2.2 (1.1, 3.5) | 280,000 (137,000, 450,000) | 3.1 (1.6, 4.6) | 398,000 (204,000, 594,000) |

This table presents the estimated number and proportion of preventable tobacco-related cancer cases under alternative tobacco control policy scenarios. Estimates were calculated under the assumption that total cancer incidence over the next 25 years (2025–2050) will be 10% lower than the GLOBOCAN projections.

Results are presented at the country level for both genders combined. The 10% increase represents a sensitivity analysis scenario to account for potential overestimation in baseline projections
