# Supplementary material for: Tobacco control policies on cancer prevention in the Eastern Mediterranean Region, 2025–2050: A modeling study
Source: PLoS Med. 2026 Apr 24;23(4):e1005032. doi: 10.1371/journal.pmed.1005032 (PMC13108767; doi:10.1371/journal.pmed.1005032)
Supplement: S11 Table — (DOCX) [file pmed.1005032.s011.docx]

**S11 Table:** Number of avoidable cancers due to highest MPOWER, a 10-unit increase in affordability index, maximizing level of literacy, and all combined policies in EMR countries (2025-2050) stratified by cancer site assuming cancer incidence is 10% lower than GLOBOCAN estimates

| Both genders | **Preventable cancer by highest MPOWER** | | **Preventable cancer by** a 10-unit **increases in tobacco affordability index** | |
| --- | --- | --- | --- | --- |
|  | PIF (95% CI) | N of cancer (95% CI) | PIF (95% CI) | N of cancer (95% CI) |
| Lung | 2.2 (1.3, 3.1) | 42,000 (25,000, 61,000) | 1.6 (1.2, 1.9) | 31,000 (24,000, 38,000) |
| Larynx | 2.1 (1.1, 3.0) | 10,000 (5,000, 14,000) | 1.5 (1.2, 1.9) | 7,000 (5,000, 9,000) |
| Esophagus | 1.2 (0.6, 1.8) | 8,000 (4,000, 12,000) | 0.8 (0.5, 1.1) | 6,000 (3,000, 8,000) |
| Pharynx | 2.9 (1.8, 3.7) | 8,000 (5,000, 11,000) | 1.7 (1.1, 2.1) | 5,000 (3,000, 6,000) |
| Oral cavity | 2.1 (1.6, 2.6) | 17,000 (13,000, 21,000) | 1.3 (1.0, 1.4) | 10,000 (8,000, 12,000) |
| Stomach | 0.5 (0.3, 0.6) | 7,000 (5,000, 9,000) | 0.4 (0.3, 0.4) | 6,000 (5,000, 6,000) |
| Colorectal | 0.2 (0.2, 0.3) | 4,000 (3,000, 5,000) | 0.2 (0.1, 0.2) | 3,000 (2,000, 4,000) |
| Liver | 0.6 (0.3, 0.8) | 12,000 (5,000, 14,000) | 0.4 (0.2, 0.5) | 8,000 (4,000, 9,000) |
| Pancreas | 0.7 (0.4, 0.9) | 4,000 (2,000, 5,000) | 0.5 (0.4, 0.6) | 3,000 (2,000, 3,000) |
| Leukemia | 0.4 (0.2, 0.5) | 3,000 (2,000, 4,000) | 0.3 (0.2, 0.3) | 2,000 (2,000, 2,000) |
| Bladder | 1.3 (0.7, 1.9) | 18,000 (10,000, 25,000) | 1.0 (0.7, 1.2) | 13,000 (9,000, 16,000) |
| Kidney | 0.5 (0.4, 0.6) | 2,000 (2,000, 2,000) | 0.4 (0.3, 0.4) | 1,000 (1,000, 2,000) |
| Cervix | 0.6 (0.5, 0.8) | 3,000 (2,000, 4,000) | 0.6 (0.5, 0.8) | 3,000 (2,000, 4,000) |
| All-Tobacco related | 1.1 (0.6, 1.5) | 139,000 (83,000, 187,000) | 0.8 (0.6, 0.9) | 98,000 (71,000, 115,000) |
|  | **Preventable cancer from maximizing literacy rate** | | **Preventable cancers from combined implementation of all policies** | |
|  | PIF (95% CI) | N of cancer (95% CI) | PIF (95% CI) | N of cancer (95% CI) |
| Lung | 3.8 (1.5, 7.5) | 74,000 (30,000, 147,000) | 6.0 (2.6, 10.3) | 128,000 (57,000, 220,000) |
| Larynx | 5.0 (2.3, 8.8) | 23,000 (11,000, 41,000) | 6.8 (3.2, 11.1) | 35,000 (16,000, 57,000) |
| Esophagus | 2.8 (1.5, 4.2) | 19,000 (10,000, 29,000) | 3.7 (2.5, 4.9) | 28,000 (19,000, 37,000) |
| Pharynx | 5.4 (5.2, 5.7) | 15,000 (15,000, 16,000) | 8.0 (4.6, 11.4) | 25,000 (14,000, 36,000) |
| Oral cavity | 5.4 (3.8, 7.0) | 43,000 (31,000, 57,000) | 6.8 (5.0, 8.6) | 60,000 (45,000, 77,000) |
| Stomach | 1.0 (0.5, 1.5) | 15,000 (7,000, 22,000) | 1.4 (0.8, 1.8) | 23,000 (14,000, 31,000) |
| Colorectal | 0.4 (0.2, 0.5) | 7,000 (3,000, 10,000) | 0.5 (0.3, 0.7) | 11,000 (7,000, 14,000) |
| Liver | 1.5 (0.5, 2.0) | 27,000 (9,000, 36,000) | 1.9 (0.5, 2.6) | 39,000 (11,000, 52,000) |
| Pancreas | 1.1 (0.3, 1.9) | 6,000 (2,000, 10,000) | 1.8 (0.9, 2.4) | 10,000 (5,000, 14,000) |
| Leukemia | 0.7 (0.3, 1.1) | 5,000 (2,000, 8,000) | 1.1 (0.6, 1.4) | 8,000 (5,000, 11,000) |
| Bladder | 3.0 (1.1, 5.1) | 40,000 (14,000, 69,000) | 4.0 (1.7, 6.1) | 59,000 (25,000, 92,000) |
| Kidney | 1.1 (0.7, 1.5) | 5,000 (3,000, 6,000) | 1.5 (0.9, 2.0) | 6,000 (4,000, 9,000) |
| Cervix | 0.0 (0.0, 0.0) | 0 (0, 0) | 0.8 (0.6, 0.9) | 4,000 (4,000, 5,000) |
| All-Tobacco related | 2.2 (1.1, 3.5) | 280,000 (137,000, 450,000) | 3.1 (1.6, 4.6) | 438,000 (225,000, 653,000) |

This table presents the estimated number and proportion of preventable tobacco-related cancer cases under alternative tobacco control policy scenarios. Estimates were calculated under the assumption that total cancer incidence over the next 25 years (2025–2050) will be 10% lower than the GLOBOCAN projections.

Results are presented by cancer site for both genders combined. The 10% increase represents a sensitivity analysis scenario to account for potential overestimation in baseline projections
