# Supplementary material for: Tobacco control policies on cancer prevention in the Eastern Mediterranean Region, 2025–2050: A modeling study
Source: PLoS Med. 2026 Apr 24;23(4):e1005032. doi: 10.1371/journal.pmed.1005032 (PMC13108767; doi:10.1371/journal.pmed.1005032)
Supplement: S12 Table — (DOCX) [file pmed.1005032.s012.docx]

**Sensitivity Analysis**

We also conducted a sensitivity analysis to assess the potential interactive effects of the simultaneous implementation of multiple policy interventions. Specifically, we included an interaction term between literacy rate and the MPOWER score in the regression models. The results indicated a statistically significant multiplicative interaction between literacy rate and MPOWER score, suggesting that the impact of tobacco control policies varies by educational attainment. The regression results for men and women are presented below:

**S12 Table**: Fixed-effects multiple linear regression examining the association between changes in tobacco smoking prevalence and policy interventions, including MPOWER score, literacy rate, and affordability index, with a multiplicative interaction between literacy rate and MPOWER score.

|  | Model A | | R^2^ | | |
| --- | --- | --- | --- | --- | --- |
| **Men** | β coefficient (95% CI) | P-value | Within | Between | Overall |
| MPOWER | -1.25 (-2.04, -0.46) |  | 0.34 | 0.06 | 0.048 |
| Affordability | -0.06 (-0.15, 0.02) | 0.151 |  |  |  |
| Literacy rate | -0.53 (-0.78, -0.28) | <0.001 |  |  |  |
| Interaction term | 0.01 (0.003, 0.02) | 0.006 |  |  |  |
| **Women** |  |  |  |  |  |
| MPOWER | -0.32 (-0.46, -0.03) | <0.001 | 0.40 | 0.01 | 0.003 |
| Affordability | -0.04 (-0.07, -0.008) | 0.013 |  |  |  |
| Literacy rate | -0.08 (-0.12, -0.03) | 0.001 |  |  |  |
| Interaction term | 0.003 (0.001, 0.004) | 0.001 |  |  |  |

β coefficients were estimated using fixed-effects panel regression models including both country (location) and year fixed effects, based on country-level data from 2010 to 2020. The dependent variable was the change in gender-specific smoking prevalence. Independent variables included changes in MPOWER implementation score, tobacco affordability index, and literacy rate. An interaction term between MPOWER score and literacy rate was also included in the model.

Each coefficient represents the change in smoking prevalence (percentage points) associated with a one-unit increase in the corresponding policy indicator in the fully adjusted model, accounting for the interaction term.

R² statistics are reported as within-country, between-country, and overall measures of model fit. P-values correspond to two-sided tests, with statistical significance defined as P < 0.05. CI = 95% confidence interval.
