# Supplementary material for: Tobacco control policies on cancer prevention in the Eastern Mediterranean Region, 2025–2050: A modeling study
Source: PLoS Med. 2026 Apr 24;23(4):e1005032. doi: 10.1371/journal.pmed.1005032 (PMC13108767; doi:10.1371/journal.pmed.1005032)
Supplement: S13 Table — (DOCX) [file pmed.1005032.s013.docx]

**S13 Table.** Projected number and proportion of preventable tobacco-related cancers under the combined policy scenario accounting for interaction between MPOWER score and literacy rate, A 25-year projection from 2025-2050 stratified by country and gender

|  | **Men** | | **Women** | |
| --- | --- | --- | --- | --- |
| Country | PIF (95% CI) | N of cancer (95% CI) | PIF (95% CI) | N of cancer (95% CI) |
| Afghanistan | 18.0 (16.0, 20.0) | 45,583 (40,465, 50,701) | 2.0 (1.5, 2.5) | 4,299 (3,198, 5,401) |
| Bahrain | 0.4 (0.0, 2.6) | 84 (2, 621) | 0.9 (0.3, 1.5) | 88 (31, 144) |
| Egypt | 1.6 (0.0, 3.6) | 32,252 (200, 71,645) | 0.4 (0.0, 0.8) | 4,239 (114, 9,386) |
| Iran | 1.2 (0.0, 3.1) | 25,987 (206, 67,093) | 1.2 (0.7, 1.8) | 16,656 (8,956, 23,984) |
| Iraq | 0.9 (0.0, 3.4) | 4,172 (138, 15,170) | 1.9 (1.3, 2.4) | 4,582 (3,094, 5,904) |
| Jordan | 0.2 (0.0, 2.6) | 347 (18, 4,671) | 0.3 (0.0, 0.8) | 268 (6, 699) |
| Kuwait | 0.4 (0.0, 2.3) | 401 (10, 2,289) | 1.2 (0.7, 1.8) | 491 (266, 716) |
| Lebanon | 0.8 (0.0, 3.3) | 691 (17, 2,954) | 0.8 (0.2, 1.8) | 535 (123, 1,169) |
| Morocco | 2.8 (0.4, 5.8) | 19,788 (2,759, 41,578) | 1.2 (0.6, 1.7) | 4,442 (2,069, 6,322) |
| Oman | 0.3 (0.0, 1.9) | 172 (6, 1,060) | 0.3 (0.0, 0.8) | 71 (0, 165) |
| Pakistan | 5.7 (3.3, 8.1) | 103,955 (60,047, 147,862) | 3.6 (3.0, 4.2) | 45,468 (37,878, 53,058) |
| Qatar | 1.1 (0.0, 3.0) | 248 (3, 654) | 0.5 (0.0, 1.0) | 49 (2, 96) |
| Saudi Arabia | 0.3 (0.0, 2.0) | 1,711 (50, 9,909) | 0.6 (0.1, 1.0) | 1,116 (226, 2,006) |
| Tunisia | 1.4 (0.1, 4.4) | 4,024 (273, 12,485) | 2.0 (1.2, 2.6) | 2,573 (1,545, 3,276) |
| United Arab Emirates | 0.4 (0.0, 2.2) | 337 (8, 1,741) | 0.9 (0.3, 1.4) | 400 (160, 641) |
| Yemen | 4.2 (2.4, 6.0) | 8,122 (4,614, 11,631) | 4.6 (4.0, 5.1) | 7,683 (6,792, 8,574) |
| EMR | 2.8 (1.2, 4.9) | 247,873 (108,816, 442,065) | 1.7 (1.2, 2.3) | 92,960 (64,460, 121,541) |

This table presents the projected number and proportion of preventable tobacco-related cancer cases under a combined policy scenario, including maximum implementation of the MPOWER package, maximized literacy rate, and a 10-unit increase in the tobacco affordability index. The model accounts for a multiplicative interaction between MPOWER score and literacy rate.

Estimates represent a 25-year projection (2025–2050) and are stratified by country and gender. Preventable fractions (PIFs) and corresponding numbers of preventable cases are presented with 95% confidence intervals (CIs).

Projections cover the 25-year period from 2025 to 2050 and are based on cancer incidence estimates from the Global Cancer Observatory (GLOBOCAN), International Agency for Research on Cancer (IARC).

Results are based on the sensitivity analysis incorporating the interaction term between MPOWER and literacy rate.

Results are presented at the country level and stratified by gender.

PIF = Potential Impact Fraction; EMR = Eastern Mediterranean Region; CI = Confidence Interval.
