# Supplementary material for: Tobacco control policies on cancer prevention in the Eastern Mediterranean Region, 2025–2050: A modeling study
Source: PLoS Med. 2026 Apr 24;23(4):e1005032. doi: 10.1371/journal.pmed.1005032 (PMC13108767; doi:10.1371/journal.pmed.1005032)
Supplement: Attachment — Submitted filename: Response_to_Reviewers.docx [file pmed.1005032.s017.docx]

Dear Editor,

We thank the reviewers for their careful evaluation of the manuscript and for their constructive comments provided. We have addressed all comments in detail below and revised the manuscript accordingly. All changes have been highlighted in the revised version of the manuscript.

King Regards,

Saeed Nemati

**Early Detection, Prevention and Infections (EPR) Branch**

**International Agency for Research on Cancer, Lyon, France**

**Email:** [**nematis@iarc.who.int**](mailto:nematis@iarc.who.int)

Comments from the reviewers:

Reviewer #1: The paper addresses an important question regarding the projected impact of tobacco control policies on cancer incidence in the Eastern Mediterranean Region (EMR). While the study is timely and well-motivated, several methodological and statistical aspects need clarification or additional justification to ensure transparency and credibility of the projections.
Major Comments

a)      The analysis implicitly assumes a causal relationship between tobacco control measures (MPOWER score, affordability index, and literacy rate) and smoking prevalence. However, the fixed-effects linear regression framework can only identify associations, not causality. This limitation should be explicitly acknowledged, with a discussion of potential time-varying confounders such as economic growth, enforcement intensity, or cultural changes.

**Response #1:** We thank the reviewer for this important comment. We added the following paragraph to Material and methods:

“It should be noted that the statistical approaches used in this study can only identify association, while we assumed a cause-and-effect relationship between previously implemented policies and concurrent changes in tobacco smoking prevalence but did not consider other potential causes of these changes.

b)      The regression models are based on six time points (2010-2020), which may not adequately capture non-linear trends or policy lag effects. The linearity assumption should be justified, and alternative specifications (e.g., random-effects, mixed, or spline-based models) explored. Moreover, the methods do not explain how uncertainty was propagated from regression coefficients through to PAF and PIF estimates. It should be clarified whether bootstrapping, Monte Carlo simulation, or the delta method was used to derive confidence intervals.

**Response #2:** Thanks for your comment. To assess linearity assumption, we explored alternative model specifications including random effect and fixed-effect model with quadratic term. The results of random effect model were consistent with our fixed-term model. Moreover, Quadratic term was not statistically significant for most main exposures, supporting the linearity assumption. It was only significant for MPOWER score in women, suggesting some degree of non-linearity.

The results of regression model with quadratic term are presented as follows for both men and women:

Table: Assessing non-linearity assumption (a quadratic relationship) between tobacco smoking prevalence and anti-tobacco policies in EMR countries stratified by gender

|  | Non-linearity assumption | |
| --- | --- | --- |
| **Men** | β coefficient (95% CI) | P-value |
| MPOWER | 0.011 | 0.435 |
| Affordability | 0.007 | 0.070 |
| Literacy rate | 0.001 | 0.481 |
| **Women** |  |  |
| **MPOWER*** | **0.011** | **0.009** |
| Affordability | 0.001 | 0.262 |
| Literacy rate | 0.0001 | 0.600 |

Uncertainty was assed using a non-parametric bootstrap procedure with 1,000 replications at the country level. The 95% confidence interval (Cis) was calculated from the empirical distributions of the bootstrap estimates.

c)      Policy interventions rarely have immediate effects. The authors should clarify whether lag terms were considered (e.g., policy effects at year t influencing smoking prevalence at t+2). Without lag adjustment, short-term associations may overestimate the projected preventive potential.

**Response #3:** We thank the reviewer for this important point regarding policy lag effects. We agree that tobacco control policies may not have immediate effect on smoking prevalence and the lagged associations are conceptually relevant. However, formal lagged regression model could not be applied due to the structure of the available data. MPOWER score, literacy rate, and affordability index were available for the years (2010, 2012, 2014, 2016, 2018, 2020), whereas smoking prevalence estimates were available for non-aligned and irregular years (2010, 2015, 2018, 2019, 2020, 2023). This temporal mismatch precluded the construction of consistent lag terms (e.g., t+2 or t+5) without substantial interpolation or imputation, which could introduce additional bias. Our results should be interpreted as the medium-term association between tobacco smoking and antitobacco policies rather than immediate effects. We have added this to limitation of our study that failure to account policy lag may lead to overestimation of short-term preventive potential.

We have added the following paragraph to limitations in order to address the potential bias due to policy lag effects:

“We assumed that the effect of improvements in policy implementation was immediate, beginning in 2025 and remaining constant over the subsequent 25 years. In practice, however, tobacco control policies are typically implemented gradually, and behavioral responses often occur with time lags. However, due to the calendar years of the available data on tobacco smoking prevalence and policy interventions in EMR countries, we were unable to apply formal lagged analyses.”

d)      Including both country and year fixed effects can control for unobserved heterogeneity but may also absorb much of the true policy variation, particularly when changes in MPOWER or literacy are slow. This limitation should be discussed, and measures of model fit (R² within, between, and overall) reported for transparency.

**Response #4:** Thanks for the insightful comment. We agree that including both country and year as fixed effects may absorb part of the true policy-related variation particularly for exposures that change slowly over time, such as MPOWER scores or literacy rates. We have now explicitly acknowledged this as a limitation in this manuscript.

For transparency, we have also reported model fit statistics for all fixed-effects models, including within, between, and overall R² values to Supplementary Table 2.

e)      Five countries were excluded due to missing data. The authors should examine whether these exclusions bias results (e.g., systematically low-HDI or high-prevalence countries). Sensitivity analyses using imputed or regional averages for missing indicators would strengthen confidence in regional estimates.

**Response #5:** Thanks for the comment. We agree that excluding five countries due to limited data may introduce selection bias, particularly when all these countries belong to lower HDI settings. However, the countries excluded from our analysis have experienced prolonged political instability and civil conflicts over the past decade, which substantially determines the reliability of cancer surveillance, monitoring tobacco smoking, and implementation of anti-tobacco policies. Consequently, available estimates of cancer incidence, smoking prevalence, MPOWER scores, and tobacco affordability in these settings are likely to be highly uncertain and not directly comparable with the rest of the region even with the low HDI countries in the region.

Moreover, due to fundamentally different political and structural conditions in these countries from other countries in the region, we believe that simple imputation approaches such as using regional or HDI-stratified averages would not adequately address the issue and could introduce additional measurement bias. Therefore, we opted to exclude these countries from the analysis to avoid measurement error. However, we acknowledge that this exclusion may lead to conservative regional estimates, which we now explicitly acknowledge as a limitation in the manuscript.

f)      The method of multiplying "policy gaps" by regression coefficients assumes constant marginal effects and independence among policies. This simplification may not hold if policies interact—for instance, literacy and affordability could jointly modify smoking behavior. A sensitivity analysis exploring non-additivity of combined policies is recommended.

**Response #6:** We thank the reviewer for this important comment regarding the assumption of additive and independent policy effects. To address this concern, we evaluated the presence of multiplicative interactions between policy measures. Our analysis identified a statistically significant interaction between literacy rate and MPOWER score, indicating that these factors jointly modify smoking behavior. Specifically, the effect of the MPOWER score was attenuated at higher literacy levels, and this effect modification was observed in both men and women. We have incorporated this analysis as a sensitivity analysis and report the corresponding regression results and estimates of preventable cancer cases in **Supplementary Tables 12–14**.

g)      GLOBOCAN projections are useful but may not reflect national registry differences or population dynamics. The authors should justify using GLOBOCAN rather than national data or age-period-cohort projections and discuss potential biases this introduces.

**Response #7:** The absence of robust population-based cancer registry data in most EMR countries limited our analysis to GLOBOCAN estimates. We acknowledge that using GLOBOCAN estimates may underestimate incidence rates for cancers such as lung and larynx in high-smoking countries like Iraq, Egypt, Kuwait, and Jordan. Lack of data from well-established population-based cancer registry prevented the use of more sophisticated methods such as Age-Period-Cohort analysis. We explicitly acknowledged these limitations in this manuscript.

h)      The use of Levin's formula assumes uniform exposure and constant relative risk across populations. Given heterogeneity in smoking intensity and patterns across EMR countries, this assumption should be acknowledged. The manuscript should also clarify whether relative risks were applied uniformly across all countries or varied by gender and cancer site (as suggested in Supplementary Table 5).

**Response #8:** We thank the reviewer for this important comment. We acknowledge that the application of Levin’s formula assumes uniform exposure within populations and constant relative risks across countries. In our analysis, relative risks were applied in a gender-specific manner and by cancer site, as reported in Supplementary Table 5.

However, country-specific relative risks by cancer site were not available for the countries included in this study. Consequently, relative risks were assumed to be constant across countries within each sex and cancer site. We recognize that smoking intensity, patterns of tobacco use, and background risk profiles may vary across EMR countries, potentially leading to heterogeneity in true relative risks.

i)      The method for deriving confidence intervals for projected cancer cases remains unclear. It should be explained whether uncertainty in smoking prevalence, relative risks, and incidence projections was jointly propagated; otherwise, reported intervals may be overly narrow.

**Response #9:** We thank the reviewer for this important comment regarding uncertainty propagation. We have now clarified the method used to derive confidence intervals for projected cancer cases in the Methods section.

Uncertainty was jointly propagated for population attributable fractions (PAFs) and cancer case counts using a non-parametric bootstrap approach. Specifically, PAF estimates and corresponding cancer counts were recalculated across 1,000 bootstrap replications, and 95% confidence intervals were derived from the empirical distributions.

Relative risks were treated as fixed parameters and were not resampled. Similarly, uncertainty in future incidence projections was not explicitly modeled. We acknowledge that, as a result, the reported confidence intervals may underestimate total uncertainty, and we have explicitly noted this limitation in the manuscript.

j)      HDI stratification is a useful approach but was implemented categorically, which may obscure within-group heterogeneity. The authors could justify the grouping scheme or test HDI as a continuous variable in sensitivity analysis.

**Response #10:** We thank the reviewer for this comment and would like to clarify that the regression analysis was conducted at country level and did not include any HDI indicator. HDI categories were used only at the results summarizing stage, where countries were grouped into four HDI levels to aggregate and present the number of potentially preventable cancer cases in a policy relevant manner. This grouping was intended for descriptive and comparative purposes rather than for effect estimation. As such, the concern regarding within-group heterogeneity does not apply to the regression analyses. We have now clarified this distinction between modeling and results presentation in the manuscript to avoid confusion.

**Minor Comments**

a)      Ensure consistent notation throughout (e.g., β₁ vs. B₁, consistent equation formatting).

**Response #1:** Thank you for the comment. We have revised the manuscript and replaced all coefficients with β for consistency.

b)      Provide a clear rationale for the 10-unit increase in the affordability index—does it reflect a realistic or policy-relevant shift?

**Response #2:** Thank you for this comment. The rationale for selecting a 10-unit increase in the affordability index was informed by benchmarking against countries with strong tobacco control performance. In most EMR countries, the affordability index remains below 5% of GDP per capita, indicating that cigarettes are relatively affordable. In contrast, in several European countries with well-established tobacco control policies, the affordability index typically ranges between 10% and 15% of GDP per capita. Based on this observed gap, we considered a 10-unit increase in the affordability index as a policy-relevant and realistic benchmark, representing the magnitude of change required for EMR countries to approach affordability levels observed in high-performing settings.

c)      Clarify whether sex-specific models were estimated separately or through interaction terms, and whether the results were statistically compared.

**Response #3:** Sex-specific models were conducted by estimating separate regression model for men and women. Difference between men and women were therefore describe qualitatively, and formal statistical comparisons were not the primary objective of the study. We have now clarified this in the Method section.

d)      Indicate if all variables were standardized before regression to aid interpretability.

**Response #4:** Smoking status variable and literacy rate were age-standardized prior to analysis, consistent with international reporting. However, no additional standardization was applied for other variables such as MPOWER score, and affordability index before regression. Policy related exposures such as MPOWER and affordability index were retained in their original measurement scales, as these variables represent policy implementation level rather than continuous biological measures. We have now clarified this in the Methos section.

e)      Specify how 2025 prevalence was derived and whether demographic projections were incorporated into subsequent estimates.

**Response #5:** The prevalence of tobacco smoking in 2025 was directly extracted from WHO repository dataset. Cancer incidence projections were obtained from GLOBOCAN, which provides estimates of the absolute number of incident cancer cases at 5-year intervals (2025, 2030, 2035, 2040, 2045, and 2050). To derive annual estimates, we calculated the difference in projected case numbers between two consecutive 5-year time points and then divided this difference by five, assuming a constant linear trend within each 5-year interval. The resulting annual increments were subsequently applied to estimate cancer incidence for intermediate years. We have now clarified this procedure in the Methods section.

f)      A directed acyclic graph (DAG) or conceptual framework linking policy → smoking → cancer would strengthen the methodological transparency.

**Response #6:** Thanks for the comment. A directed acyclic graph is added to the manuscript to explain conceptual framework of the study.

g)      Sensitivity analyses using alternative relative risks or cancer projections (e.g., varying GLOBOCAN assumptions by ±10%) would enhance robustness.

**Response #7:** Sensitivity analyses incorporating two alternative cancer projection scenarios were added. In these analyses, we assumed that future cancer incidence projections were **10% higher and 10% lower than the GLOBOCAN estimates**, to assess the robustness of our findings to uncertainty in cancer burden projections. Results could be found in supplementary materials (Tables 8-11)

h)      Quantify uncertainty in cancer incidence projections due to possible under-reporting in registry data.

**Response #8:** We have already acknowledged the lack of cancer registry data in EMR countries as a limitation of our study. In the absence of such data from EMR countries, quantifying uncertainty in cancer incidence projections due to underreporting is not possible.

i)      Consistently describe scaling of variables: for example, clarify whether a higher affordability index implies higher or lower affordability.

**Response #9:** We clarify that higher values of the affordability index correspond to lower cigarette affordability. The index is measured as the proportion of GDP per capita required to purchase cigarettes; therefore, an increase in the index indicates that cigarettes become more expensive relative to income.

j)      Explain the MPOWER scoring system and interpret the meaning of a one-unit change in the regression context.

**Response #10:** We added the following paragraph as the explanation on MPOWER scoring system and the interpretation of each unit increase in the context of regression.

“While the maximum score for the ‘M’ component is 4, the other six components each have a maximum score of 5, resulting in an overall MPOWER score ranging from 7 to 34.”

“Regression coefficients of MPOWER represent percentage change of tobacco smoking associated with one-unit change in MPOWER score.”

k)      Check for multicollinearity among policy variables (e.g., literacy and MPOWER) using VIF or correlation analysis, and report results.

**Response #11:** Multicollinearity among covariates was assessed using variance inflation factors (VIFs). All VIF values were close to 1 (range: 1.06–1.22; mean VIF = 1.15), indicating **no evidence of problematic multicollinearity**. We have now reported this assessment in the Methods section.

l)      Clarify whether regressions were population-weighted; unweighted models may overrepresent small countries.

**Response #12:** The regression models were not population-weighted. Given the ecological study design, each country was treated as a single unit of observation with equal contribution to the analysis. This approach was used to examine country level association rather than population-level effect and to avoid disproportionate influence of highly populated countries. We have now clarified this modeling choice in the methods section,

m)      Note that projecting linear effects to 2050 assumes policy impacts remain stable over 30 years—this should be explicitly acknowledged as a limitation.

**Response #13:** We now explicitly acknowledge the assumption of stable policy effects over the projection period as a limitation.

n)      Ensure all acronyms (MPOWER, PAF, PIF) are defined at first mention.

**Response #14:** We confirm that all acronyms, including **MPOWER**, **PAF**, and **PIF**, have been defined at their first mention in the manuscript.

o)      Discuss data harmonisation across sources (WHO, World Bank, IARC) and potential measurement inconsistencies.

**Response #15:** Data were harmonized at the country–year level. Differences in reporting years across sources were addressed by converting calendar years into measurement waves. Residual measurement inconsistencies are acknowledged as a limitation.

p)      Justify using the World Bank's HDI instead of the standard UNDP classification.

**Response #16:** Thanks for the comment. We changed it to UNDP classification.

q)      Ensure the equations and tables display appropriate spacing and consistent notation.

**Response #17:** We have reviewed all equations and table and ensured consistent notation and appropriate spacing throughout the manuscript. Necessary formatting revisions have been applied.

r)      Review confidence intervals in Tables 1-2, which appear narrower than expected given multi-step uncertainty; verify computation.

**Response #18:** We have carefully reviewed the confidence intervals reported in Tables 1 and 2 and verified the underlying computations.

s)      Temper the causal interpretation that "improving literacy offers greater benefit than MPOWER," as differing mechanisms and potential biases limit comparability.

**Response #19:** Thanks for the comment. We have now revised the wording as follows:

Strengthening tobacco control policies particularly improving literacy in low-HDI countries may potentially contribute to reductions in future cancer burden in EMR.

Reviewer #2: Overall I find this an interesting paper and highly relevant to current global policy priorities. My comments are below:

1)      Make it clear that "increase in affordability index" means lower affordability and give a brief sentence explaining how it is measured for readers' convenience to aid interpretation of your findings.

**Response #1:** We clarify that higher values of the affordability index correspond to lower cigarette affordability. The index is measured as the proportion of GDP per capita required to purchase cigarettes; therefore, an increase in the index indicates that cigarettes become more expensive relative to income.

2)      Your analysis appears to assume immediate effect of policy post 2025. First, confirm whether this is the case, and second, perhaps discuss how this is an optimistic scenario and how tobacco control policies are often phased in adoption and implementation.

**Response #2:** We clarify our projection assumes that full implementation of anti-tobacco policies begins in 2025 and their effects of prevalence of tobacco smoking and cancer reduction will be without delay. We also confirm that full implementation of anti-tobacco policies with immediate impact on tobacco smoking and cancer reduction is very optimistic, meanwhile in practice tobacco control policies are typically phased in gradually, and behavioral responses often occur with time lags. We have now clarified this assumption and discussed it as a limitation in the Discussion.

3)      Perhaps you may consider analysing multiple scenarios, including more realistic scenarios. For example you could take one country from 2000 to 2025 with a more "successful" implementation of MPOWER and apply that (i.e. what could other countries realistically achieve if they emulated this specific country?) to the others.

**Response #3:** We truly appreciate this insightful comment. We explored feasibility of benchmark-based scenarios using empirically observed MPOWER improvement. However, the largest observed MPOWER score improvement over the past decade in the region was 9 points which was observed in Qatar and Saudi Arabia. Appling such an improvement across countries would result in most EMR countries reaching or exceeding the maximum MPOWER score (34), leading to a pronounced ceiling effect. As a result, the benchmarking based on the improvement observed in the most successful country in the region will provide similar results as our full-implementation scenario. We therefore retained the full MPOWER implementation as the upper bound estimates and now explicitly acknowledge this consideration in our discussion. We also explored the feasibility of benchmark-based scenarios for the affordability index. We found that the largest observed increases in the affordability index were **38 units in Yemen** and **14 units in Iran**, representing the most pronounced changes in the region. These magnitudes are substantially larger than the **10-unit increase scenario**, suggesting that a benchmark-based approach would exceed or closely approximate the upper-bound assumptions already considered in our analyses.

4)      Explain how you calculated confidence intervals for your PIFs. You may consider use of Markov chain analysis to obtain confidence intervals.

**Response #4:** We thank the reviewer for this important comment regarding uncertainty propagation. We have now clarified the method used to derive confidence intervals for projected cancer cases in the Methods section.

Uncertainty was jointly propagated for population attributable fractions (PAFs) and cancer case counts using a non-parametric bootstrap approach. Specifically, PAF estimates and corresponding cancer counts were recalculated across 1,000 bootstrap replications, and 95% confidence intervals were derived from the empirical distributions.

Relative risks were treated as fixed parameters and were not resampled. Similarly, uncertainty in future incident projections was not explicitly modeled. We acknowledge that, as a result, the reported confidence intervals may underestimate total uncertainty, and we have explicitly noted this limitation in the manuscript.

5)      You may consider discussing latency effects (although you touch on this at the end of the manuscript by hinting at the possibility of conducting age-period-cohort analyses) in the association between smoking and cancer outcomes. For example, preventing uptake among younger people will only result in reductions in mortality decades later as the cancer outcomes you analysed take many years to develop when caused by smoking.

**Response #5:** Thank you for this comment. We have now clarified in the Limitations that projections in this study are based on an optimistic scenario assuming an immediate impact of tobacco control policy measures on smoking prevalence and, consequently, on cancer incidence. In practice, reductions in tobacco exposure, particularly those achieved through exposure prevention, often require one or more decades to translate into measurable reductions in cancer incidence and mortality due to the long latency of smoking-related cancers. We have now expanded our limitation in this regard.

6)      I suggest reviewing and discussing other literature relevant to other WHO regions, in particular whether other researchers have attempted projecting impact of tobacco control policies, how your methods and findings may have differed, and whether there are any strengths or limitations of your approach compared with other studies.

Response #6: Thanks for the comment. We have now added the following paragraph to discuss different approaches used to investigate effect of policy implementation on prevalence of tobacco smoking in other regions such as Europe and the US.

“In the present study, we used a fixed-effects panel regression framework to examine the association between changes in tobacco control policy implementation and levels of smoking prevalence.

In contrast, several previous studies particularly from European settings have modeled relative changes in smoking prevalence as a function of changes in policy implementation scores. While informative for short-term behavioral responses, such approaches can be more sensitive to short-term fluctuations and the choice of baseline prevalence. Our level-based modeling strategy provides more stable estimates, is less sensitive to baseline choice, and is better suited to irregular panel data structures, which are common in the EMR context. In other regions, particularly the United States, more advanced simulation-based approaches such as smoking history generator models have been used to project the long-term impact of tobacco control policies on smoking prevalence and health outcomes. Although these models can generate highly detailed and potentially more precise projections, they require comprehensive longitudinal smoking profiles, including age- and sex-specific prevalence by smoking status (never, current, former), age at initiation, cessation rates, and smoking intensity. Such detailed data are not consistently available for most EMR countries.”

7)      Literacy may not be a confounder for the association between tobacco control policies and smoking prevalence, or simply a direct determinant. Literacy may also interact with MPOWER policies, acting as a moderator of policy effectiveness (for example literacy may represent a proxy for health literacy, which in turn influences the effectiveness of tobacco control information campaigns at changing behaviour). Perhaps you may briefly discuss how and whether the association between literacy and smoking prevalence is causal, and potential pathways.

**Response #7:** We did not model literacy as a confounder in the causal pathway between tobacco control policies and smoking prevalence. Rather, we explicitly treated literacy as a factor associated with smoking prevalence. The following paragraph in the discussion we have tried to explain how increasing literacy rate might lead to reduction in prevalence of tobacco smoking and consequently cancer incidence. We also expanded our discussion to the association between literacy rate and better implementation of MPOWER domains such as understanding health messages, and higher likelihood of quitting smoking. “In our study, we demonstrated that maximizing literacy rate could be the most effective strategy particularly for countries with medium and low HDI, that may lead to the potential prevention of 1.5%, and 5.0% of all incident tobacco-related cancers, respectively, in these countries by 2050. By improving education, populated countries with low literacy rates (such as Afghanistan, Pakistan, and Yemen) could potentially achieve a notable decrease in smoking prevalence that could lead to the prevention of a significant number of cancers cases. **While increasing literacy is not a targeted anti-tobacco policy,** its strong inverse correlation with smoking prevalence has been documented in numerous studies **Higher education may facilitate better access to health information and increase the public awareness of the harmful effects of tobacco use messages are more likely to be understandable to and followed by people with higher education Higher education is also associated with being more aware of the risks of smoking and increases the likelihood of quitting or avoiding smoking** Maximizing literacy rates has broader benefits for cancer control beyond just reducing tobacco use, undermining the importance of increasing literacy rate in the context of low- and middle-income countries. In such countries, higher education levels additionally lead to a reduction in exposure to other cancer risk factors and improved earlier cancer diagnosis through timely medical referrals. We have also added a Directed Acyclic Graph (DAG) illustrating the relationships among policy interventions, tobacco smoking prevalence, and cancer incidence.

8)      You may speculate on whether individual MPOWER domains may be driving changes in smoking prevalence more than others.

**Response #8:** Thank you for this comment. We acknowledge that individual MPOWER domains may contribute differently to changes in smoking prevalence. However, examining domain-specific effects was beyond the primary scope of the present study, which focused on the combined impact of MPOWER implementation as a comprehensive policy package on cancer incidence in EMR countries.

Importantly, this question has been explicitly addressed in our previous study published in *PLOS ONE*, where we examined the associations between individual MPOWER components and smoking prevalence. In that study, we found that among men, health warnings, bans on smoking in public places, and tobacco taxation showed the strongest associations with reductions in smoking prevalence, whereas among women, bans on smoking in public places, bans on tobacco advertising, and health warnings were the leading MPOWER domains associated with changes in smoking prevalence. More details could be found here (PMID: 38394166)

9)      You may discuss the strengths and limitations of studies/sources of RR estimates used in your analysis (e.g. biases, study design limitations, generalisability to EMR etc).

**Response #9:** Thanks for the comment.

We have now discussed the strengths and limitations of the relative risks used in this study as follows:

“Furthermore, while country-specific relative risks by cancer sites were not available for EMR countries, we used site-specific and gender-specific relative risks that were driven from high-quality systematic reviews and meta-analyses that primarily included cohort and case-control studies conducted in various regions of the world and were adjusted for potential confounders.”

10)     Likewise, are there any limitations as to how MPOWER scores are estimated?

**Response #10:** We thank the reviewer for this comment. We acknowledge that MPOWER scores have inherent limitations. Although MPOWER scores may not fully capture the extent or quality of real-world implementation of anti-tobacco policies, they are widely regarded as the most comprehensive and standardized indicators of countries’ adoption of the WHO Framework Convention on Tobacco Control (FCTC) and related tobacco control measures. We have now explicitly discussed these limitations in the manuscript.

11)     You model also seems to assume the association between affordability and smoking prevalence is linear; you may consider fitting a quadratic term in your model to asses this or briefly comment on this in the discussion.

**Response #11:** We thank the reviewer for this helpful suggestion. To assess the potential non-linearity in the association between cigarette affordability and smoking prevalence, we conducted a sensitivity analysis including a quadratic term for MPOWER, affordability, and literacy rate in the fixed-effects regression models.

The quadratic term was not statistically significant, and the estimated linear association remained stable, suggesting no strong evidence of non-linearity within the observed range of affordability. We have also evaluated the quadratic term for both MPOWER and literacy level for both men and women. For education the linear association remained stable. In contrast, for MPOWER score the quadratic term was statistically significant specifically for women indicating that marginal effect of MPOWER diminishes at higher level of policy implementation. The results of regression model with quadratic term are presented as follows for both men and women:

Table: Assessing non-linearity assumption (a quadratic relationship) between tobacco smoking prevalence and anti-tobacco policies in EMR countries stratified by gender

|  | Non-linearity assumption | |
| --- | --- | --- |
| **Men** | β coefficient (95% CI) | P-value |
| MPOWER | 0.011 | 0.435 |
| Affordability | 0.007 | 0.070 |
| Literacy rate | 0.001 | 0.481 |
| **Women** |  |  |
| **MPOWER*** | **0.011** | **0.009** |
| Affordability | 0.001 | 0.262 |
| Literacy rate | 0.0001 | 0.600 |

12)     In your introduction you mention alternative forms of tobacco products such as water pipes. Are there included in the smoking prevalence estimates? Could excluding these products or not disaggregating them from cigarettes be a limitation of your study? For example do these have different RRs for their associations with different cancers?

**Response #12:** We acknowledge that a proportion of the observed prevalence may reflect the use of other tobacco products, such as waterpipe smoking, which may be associated with different relative risks for certain cancer sites compared with cigarette smoking. However, findings from our previous study in the EMR showed that only about 1% of cancer cases were attributable to waterpipe smoking **(PMID: 35895382).** Therefore, using tobacco smoking may not impose significant effect on our estimates, while it has considered all types of tobacco smoked in the region.

We have now explicitly discussed these limitations in the manuscript.

13)     Please specify the age ranges for your tobacco use prevalence estimates by country.

**Response #13:** Thanks for your comment. We used age-standardized prevalence estimates for tobacco smoking for adults **aged ≥15 years** across countries. This information has now been explicitly clarified in the Methods section of the manuscript.
