# Supplementary material for: Tobacco control policies on cancer prevention in the Eastern Mediterranean Region, 2025–2050: A modeling study
Source: PLoS Med. 2026 Apr 24;23(4):e1005032. doi: 10.1371/journal.pmed.1005032 (PMC13108767; doi:10.1371/journal.pmed.1005032)
Supplement: Attachment — Submitted filename: Response Letter.docx [file pmed.1005032.s018.docx]

**Response to Reviewers and Editors**

**Dear Dr. Tosun,**

Thank you very much for your thoughtful comments and for the reviewers’ insightful feedback. In this revised version, we have carefully addressed all scientific and editorial concerns raised in the previous decision letter. We hope that the revisions meet the standards of PLOS Medicine for publication and adequately address all concerns raised. We would be grateful for any further suggestions that may help improve the manuscript.

Sincerely

Saeed Nemati,

on behalf of all authors

**Comments from Reviewers:**

Reviewer #1: Thank you for thoroughly addressing all my comments and for providing detailed clarifications in the revised manuscript. I appreciate the considerable effort you have invested in enhancing the rigor and transparency of the analyses. The following are additional minor comments that may further improve clarity and interpretation of the results:

a) While regression coefficients are interpreted as percentage changes in smoking prevalence, it would be helpful to briefly contextualise these effects in absolute prevalence terms (e.g., percentage-point changes). This may aid policy interpretation, particularly when translating reductions in smoking prevalence into projected cancer cases.

**Response 1:** Smoking prevalence was modelled as an absolute percentage (0–100), and regression coefficients therefore represent absolute percentage-point changes associated with a one-unit increase in the independent variables. We have now explicitly specified it in the methodology.

b) MPOWER scores, literacy rates, and affordability indices are subject to measurement error and imperfect policy enforcement. The authors may wish to acknowledge that classical measurement error in these explanatory variables could attenuate estimated associations, potentially resulting in conservative estimates of policy impact.

**Response 2:** We thank the reviewer for this insightful comment. We have revised the manuscript to more explicitly discuss potential measurement error in the explanatory variables, as detailed below:

In addition, MPOWER scores, literacy levels, and affordability indices may be affected by measurement error and may not fully reflect the real-world implementation or enforcement of policies. Such classical measurement error in the explanatory variables is likely to bias effect estimates toward the null, resulting in potentially conservative assessments of policy impact on smoking prevalence and cancer prevention.

c) Although already implicit, the manuscript would benefit from a short, explicit statement noting that ecological associations at the country level may not directly reflect individual-level causal mechanisms (i.e., the ecological fallacy), particularly when linking policy measures to smoking behaviour and cancer outcomes.

**Response 3:** We thank the reviewer for this important comment. We have now added a brief and explicit statement in the Discussion addressing the risk of cross-level inference and ecological fallacy. The revised text reads as follows:

“As with any ecological study, our findings are subject to ecological fallacy. The observed country-level associations should therefore not be interpreted as evidence of individual-level causal relationships between policy measures, smoking behaviour, and cancer outcomes.”

d) It would be useful to clarify whether projected reductions in smoking prevalence were constrained to avoid implausible negative prevalence values in countries with low baseline smoking rates under combined policy scenarios.

**Response 4:** Thanks for the comment. We have now clarified it in the material and method section as follows:

To avoid implausible negative prevalence values in low-prevalence settings, projected smoking prevalence was constrained to a minimum value of zero in all alternative scenarios.

e) The manuscript could benefit from a brief conceptual distinction between statistical uncertainty (captured via the bootstrapping procedure) and policy implementation uncertainty (e.g., feasibility, compliance, and enforcement), which is not formally modelled but is important for interpretation of the findings.

Response 5: We thank the reviewer for this insightful comment. We have now clarified in the Discussion the distinction between statistical uncertainty quantified through the bootstrapping procedure and policy implementation uncertainty, which was not formally modelled. The following text has been added:

Our analysis quantified statistical uncertainty using a bootstrapping approach. However, the reported confidence intervals do not capture uncertainty related to real-world policy implementation, including feasibility, compliance, political commitment, and enforcement capacity. Consequently, the actual effects of policy implementation may differ from model projections depending on the extent to which real-world implementation aligns with the hypothetical policy scenarios modelled in this study.


Reviewer #2: I believe that the authors have adequately addressed the reviewers' comments within the scope of this paper, and that the changes made to the manuscript have significantly improved it.

I would only make two final optional comments:

1) You said "Our analysis identified a statistically significant interaction between literacy rate and MPOWER score, indicating that these factors jointly modify smoking behavior. Specifically, the effect of the MPOWER score was attenuated at higher literacy levels...". Are you able to explain why your model may have shown this finding? Is this counterintuitive? Can this result be contextualised by findings of previous research?

**Response 1:** We thank the reviewer for this thoughtful comment. Our sensitivity analysis identified a significant interaction between MPOWER score and literacy rate. Specifically, the association between MPOWER implementation and smoking prevalence was attenuated in countries with higher literacy levels.

Several plausible explanations may account for this finding. First, countries with higher literacy rates often already have relatively higher baseline MPOWER scores, leaving less room for relative improvement in policy strength over time. In contrast, countries with lower baseline MPOWER scores may lack one or more core components of the MPOWER framework, such that incremental policy improvements could yield larger marginal effects.

Second, in settings where MPOWER scores are already relatively high, further reductions in smoking prevalence may depend more on compliance, enforcement, and broader social norms rather than the formal presence of additional policy components. Thus, improvements in MPOWER scores alone may translate into smaller observable changes in prevalence in high-literacy contexts.

Moreover, different components of the MPOWER framework may have heterogeneous effects on smoking prevalence. Previous studies have consistently shown that taxation has the strongest impact on reducing tobacco use. However, taxation interacts with broader socioeconomic conditions, including affordability and income growth. In more socioeconomically developed countries, increases in income may partially offset the impact of tax increases on tobacco affordability, potentially attenuating policy effects. This may be particularly relevant in more developed countries within the EMR where substantial economic growth has been observed over the past decade.

Some previous European studies have explored the joint effects of tobacco control policies and socioeconomic indicators on smoking prevalence. However, these studies used different statistical approaches and relied on proxies such as the Human Development Index rather than literacy rate, limiting direct comparability with our findings.

While a more detailed mechanistic investigation of this interaction is beyond the scope of the present study, our results suggest that the effectiveness of MPOWER implementation may be context-dependent and influenced by baseline socioeconomic conditions, including literacy level. Given the scope and focus of this manuscript, we believe that an in-depth investigation of this interaction would be more appropriately addressed in a separate, dedicated analysis.

2) Regarding my original comment #3 (i.e. modelling "more realistic scenarios") I believe your approach makes sense. However, another approach my had in mind was to select a country that could be considered a "leader" in the Region (say Qatar as an example) and that in this scenario all countries' MPOWER scores are raised to that of the "leader" to address the question of what if all countries of the Region emulated the example of best practice set by this leading country, and then later discuss which MPOWER domains most countries should address to catch up with the "leader".

**Response 2:** We thank the reviewer for this thoughtful and constructive suggestion. In the Eastern Mediterranean Region, the country with the highest MPOWER score is Iran, with a most recently reported score of 31, which is already close to the maximum achievable MPOWER score. Given the relatively small gap between this leading country and the theoretical maximum, a scenario in which all countries’ MPOWER scores are raised to the level of the regional leader would yield projections that are largely comparable to our existing maximum implementation scenario. For this reason, we believe that introducing an additional “regional leader” scenario would provide limited incremental insight beyond the full implementation scenario already presented. To maintain clarity and avoid redundancy in the presentation of results, we have retained the current modelling framework, which reflects the upper bound of achievable MPOWER implementation across countries.

**Requests from Editors:**

Please note that your point-by-point response did not address the list of editorial requests included in the prior decision letter. Please consider these points when revising your manuscript. In your response to this decision letter, please address the points under "OBSERVATIONAL STUDIES" and "MODELLING STUDIES" from the previous letter.

Response: We thank the editors for these comments. This study is a modelling study using secondary aggregated data; therefore, several STROBE items related to individual-level observational study design are not applicable.

We have also clarified the study design in the Abstract and Methods section as a modelling study using secondary, country-level data. We also added a sentence explaining that the data, study inputs, assumptions, analytical approaches, and results are presented based on the best practice for modeling studies.

GENERAL
* Please confirm that your title complies with to PLOS Medicine's style. Your title must be nondeclarative and not a question. It should begin with main concept if possible. "Effect of" (or “Impact of”) should be used only if causality can be inferred, i.e., for an RCT. Please place the study design ("A randomized controlled trial," "A retrospective study," "A modelling study," etc.) in the subtitle (ie, after a colon).

Response:
We thank the editors for this comment. In accordance with PLOS Medicine’s title requirements, we have revised the title to remove causal language and to include the study design in the subtitle. The revised title now reads:

**“Tobacco Control Policies and Cancer Prevention in the Eastern Mediterranean Region, 2025–2050: A Modelling Study.”**

* Statistical reporting: Please revise throughout the manuscript, including tables and figures.
- Please report statistical information as follows to improve clarity for the reader, ""XX% (95% CI [XX,YY]; p</=)"".

**Response 1:** We confirm that all statistical results throughout the manuscript, including tables and figures, have been revised and are now reported in the recommended format: XX% (95% CI [XX, YY]; p ≤ 0.05), where applicable.

- Please separate upper and lower bounds with commas instead of hyphens as the latter can be confused with reporting of negative values.

**Response 2:** We can confirm all the lower and upper bounds are separated by commas throughout the text, and tables.

- Please repeat statistical definitions (HR, CI etc.) for each set of parentheses.

**Response 3:** We confirm that all statistical abbreviations (e.g., HR, CI, RR, PIF) are now explicitly defined in each table figure legend.

* Please ensure that all abbreviations are defined at first use throughout the text (including statistical abbreviations).

**Response 4:** We can confirm that all abbreviations are defined at firs use throughout the text.

* Please ensure that tables and figures, including those in supplementary files, are appropriately referenced in the main text.

**Response 5:** We can confirm that all main and supplementary tables and figures are appropriately references in the main text.

* Please review your text for claims of novelty or primacy (e.g. 'for the first time' or ‘novel’) and remove this language.

**Response 6:** We removed these claims of novelty from the manuscript.

* Please confirm that any use of statistical terms (such as trend or significant) are supported by the data, and if not please remove them. The term trend should be used only when the test for trend has been conducted.

**Response 7:** We can confirm that statistical terms such as trend or significant were only used when they were supported by proper statistical test.

* Please provide titles and legends for all figures and tables (including those in Supporting Information files).

**Response 8:** We can confirm that all tables and figures have title and legends.

* Please define all acronyms used in each figure or table in the corresponding legend.

**Response 9:** We can confirm that all acronyms used in each figure and Table are defined in their corresponding legend.

* Please include all relevant links and access details in the Data Availability Statement. A reference to the Appendix is not sufficient.

Response 10: The full analytical code and processed datasets required to reproduce the findings of this study are publicly available on GitHub (https://github.com/nematisaeed17-lab/EMR_tobacco_policy_model/tree/016e882f636f585024c74582132a1f75b4bb2ca4) and archived in Zenodo (DOI: 10.5281/zenodo.18701105).

* The terms gender and sex are not interchangeable (as discussed in <https://www.who.int/health-topics/gender#tab=tab_1> ); please use the appropriate term.

**Response 11:** We confirm that the term “sex” has been used consistently throughout the manuscript, including in the main text, tables, and figures, as the analyses refer to biological classification (male/female) rather than gender

* We expect all researchers with submissions to PLOS in which author-generated code underpins the findings in the manuscript to make all author-generated code available without restrictions upon publication of the work. In cases where code is central to the manuscript, we may require the code to be made available as a condition of publication. Authors are responsible for ensuring that the code is reusable and well documented. Please make any custom code available, either as part of your data deposition or as a supplementary file. Please add a sentence to your data availability statement regarding any code used in the study, e.g. "The code used in the analysis is available from Github [URL] and archived in Zenodo [DOI link]" Please review our guidelines at <https://journals.plos.org/plosmedicine/s/materials-software-and-code-sharing> and ensure that your code is shared in a way that follows best practice and facilitates reproducibility and reuse. Because Github depositions can
be readily changed or deleted, we encourage you to make a permanent DOI'd copy (e.g. in Zenodo) and provide the URL.

**Response:** The full analytical code and processed datasets required to reproduce the findings of this study are publicly available on GitHub (https://github.com/nematisaeed17-lab/EMR_tobacco_policy_model/tree/016e882f636f585024c74582132a1f75b4bb2ca4) and archived in Zenodo (DOI: 10.5281/zenodo.18701105).

ABSTRACT
* Please confirm that your abstract complies with our requirements, including providing all the information relevant to this study type <https://journals.plos.org/plosmedicine/s/submission-guidelines#loc-abstract>

**Response 1:** We can confirm that our abstract complies with the journal requirements.

* Please confirm that all numbers presented in the abstract are present and identical to numbers presented in the main manuscript text.

**Response 2:** We can confirm that the numbers in the abstract are present and identical to numbers presented in the main manuscript text and tables.

* In the last sentence of the Abstract Methods and Findings section, please describe the main limitation(s) of the study's methodology.

**Response 3:** We added the main limitation of the current study to the abstract.

* In the abstract, please include the important dependent variables that are adjusted for in the analyses (if applicable).

**Response 4:** Not applicable.

* Please ensure that all abbreviations used are defined at first use.

**Response 5:** We confirm that all abbreviations used are defined at first use.

* Please provide a brief explanation what the cigarette affordability index is or means (e.g.: higher values of the affordability index equal lower cigarette affordability).

**Response 6:** We added a brief explanation for the cigarette affordability index as follows:
Higher values of the affordability index indicate that cigarettes are less affordable

* Please mention that “maximized literacy rates” means 100%.

**Response 7:** We added (100% adult literacy) to the text in the abstract.

* Please provide CI values for all numerical results presented.

**Response 8:** We added 95% CI values for all numerical results presented.

AUTHOR SUMMARY
* Please include a short, non-technical Author Summary of your research to make findings accessible to a wide audience that includes both scientists and non-scientists. The Author Summary should immediately follow the Abstract in your revised manuscript. This text is subject to editorial change and should be distinct from the scientific abstract. Ideally each sub-heading should contain 2-3 single sentence, concise bullet points containing the most salient points from your study. In the final bullet point of 'What Do These Findings Mean?', please include the main limitations of the study in non-technical language. Please see our author guidelines for more information: <https://journals.plos.org/plosmedicine/s/revising-your-manuscript#loc-author-summary>.

**Response 1:** We have added a short, non-technical Author Summary immediately following the Abstract, structured according to the journal’s guidelines.

INTRODUCTION
* “in more than 13 anatomical sites”. – We think it’d be useful to list the specific cancer types here.

**Response 1:** We added the specific cancer site associated with tobacco smoking to introduction.

* “While the maximum score for the ‘M’ component is 4, the other six components each have a maximum score of 5, resulting in an overall MPOWER score ranging from 7 to 34 (13).” – Please include this information in the Methods section.

**Response 2:** We transferred the mention part to the Method section.

METHODS AND RESULTS
* Please remove all numbering from headers.

**Response 1:** All numbers were removed from headers.

* “aged older than 15” – when reporting age, please add a unit, such as ‘years’.

**Response 2:** We have now added unit to the age as follows: “adults aged ≥15 years”

* “We also evaluated the presence of multiplicative interactions between policy measures. Our analysis identified…” – Please report findings of the sensitivity analyses in the Result section, not the Methods.

**Response 3:** Thank you for this comment. We have moved the description and findings of the sensitivity analysis from the Methods section to the Results section, as suggested.

* “Increasing the tobacco affordability index, as a second alternative scenario, was projected to prevent 0.8% (95% CI: 0.6 – 0.9) of all incident tobacco-related cancers in the EMR.” – please provide absolute numbers as well.

**Response 4:** We have now added the absolute numbers as suggested.

* Figure 2 and 3: The figures require revision:
1) Please ensure that the error bars do not overlap with the numbers on the graph.
2) Please provide a description and unit for the x-axis.
3) In the figure description, please define the meaning of the error bar.

**Response 5:** All requested modifications have been implemented in Figures 2 and 3.

* Figure 4: The figure requires revision:
1) Please provide a unit for the y-axis.
2) Please note that there’s a typo in “Medium HDI”.
3) Please include the error bars and please define their meaning in the figure description.
4) In the figure description, please provide definitions of low, medium and high HDI.
5) Does during the next 25 years mean in 25 years (i.e. in 2050)?

**Response 6:** All requested modifications have been implemented in Figures 4.

Thanks for the comments. We have modified the figure title as follows:

**Figure 4. Estimated proportion of preventable tobacco-related cancers under each prevention policy scenario, stratified by Human Development Index of EMR countries, 2025–2050.**

* Please confirm that you specified the variables controlled for in all relevant Tables.

**Response 7:** We can confirm that the variables controlled wherever they were required.

* Please confirm that you provided the unadjusted comparisons as well as the adjusted comparisons in all relevant Tables (if applicable).

**Response 8:** We can confirm that we have provided both adjusted and non-adjusted comparisons wherever they were required.

DISCUSSION
* “Additionally, the relative risks used for lung cancer in the EU were significantly higher than those estimated in Europe (33).” – please clarify.

**Response 1:** Thank you for pointing this out. We apologize for the lack of clarity. This was a wording error. Our initial intention was to compare relative risk used in Europe versus those we applied in our current analysis for EMR countries. We have now modified the text as follows:

“Additionally, the relative risks used for lung cancer in European population were higher than those applied in the present analysis for EMR countries

* Please refer to high income countries rather than "developed" or "Western" countries.

**Response 2:** We changed them to high-income countries.

* Please remove the 'conclusions' subheading from the discussion. Please also remove any other subheadings from the discussion.

**Response 3:** We removed all subheading from the discussion/

------------------------------------------------------------
General Editorial Requests

1) We ask every co-author listed on the manuscript to fill in a contributing author statement. If any of the co-authors have not filled in the statement, we will remind them to do so when the paper is revised. If all statements are not completed in a timely fashion this could hold up the re-review process. Should there be a problem getting one of your co-authors to fill in a statement we will be in contact. YOU MUST NOT ADD OR REMOVE AUTHORS UNLESS YOU HAVE ALERTED THE EDITOR HANDLING THE MANUSCRIPT TO THE CHANGE AND THEY SPECIFICALLY HAVE AGREED TO IT.

2) Please ensure that the paper adheres to the PLOS Data Availability Policy (see <http://journals.plos.org/plosmedicine/s/data-availability)>, which requires that all data underlying the study's findings be provided in a repository or as Supporting Information. For data residing with a third party, authors are required to provide instructions with contact information for obtaining the data. PLOS journals do not allow statements supported by "data not shown" or "unpublished results." For such statements, authors must provide supporting data or cite public sources that include it.

**Response 2:** The Data Availability Statement has been revised in accordance with the journal’s instructions. All code and datasets used in this study are publicly available on GitHub.

3) Please review your reference list to ensure that it is complete and correct. If you have cited papers that have been retracted, please include the rationale for doing so in the manuscript text, or remove these references and replace them with relevant current references. Any changes to the reference list should be mentioned in the rebuttal letter that accompanies your revised manuscript.

**Response 3:** We can confirm that there is no retracted paper in our reference list.

__________________________________________________
In compliance with data protection regulations, you may request that we remove your personal registration details at any time.  (Use the following URL: <https://www.editorialmanager.com/pmedicine/login.asp?a=r)>. Please contact the publication office if you have any questions.
